# Isolation of New Strains of Lactic Acid Bacteria from the Vaginal Microbiome of Postmenopausal Women and their Probiotic Characteristics

**DOI:** 10.1007/s00284-024-04034-8

**Published:** 2025-01-09

**Authors:** Indrajeet Barman, Hoonhee Seo, Sukyung Kim, Md Abdur Rahim, Youjin Yoon, Mohammed Solayman Hossain, Md Sarower Hossen Shuvo, Ho-Yeon Song

**Affiliations:** 1 Department of Microbiology and Immunology, School of Medicine, Soonchunhyang University, Cheonan-si, Chungnam 31151 Republic of Korea; 2 Human Microbiome Medical Research Center, Soonchunhyang University, Asan-si, Chungnam 31538 Republic of Korea

## Abstract

Lactic acid bacteria (LAB), traditionally consumed as fermented foods, are now being applied to the medical field beyond health-functional food as probiotics. Therefore, it is necessary to continuously discover and evaluate new strains with suitable probiotic characteristics, mainly focusing on safety. In this study, we isolated eight new strains from postmenopausal vaginal fluid using culturomics approaches, an emerging area of interest. Data showed that most strains possessed significant cell surface hydrophobicity (≥ 76%), auto-aggregation capacity (17 to 61%), strong adhesion activity (8 to 34%), and excellent resistance to gastric acid, bile salt, and digestive enzyme, enhancing their survival in the gastrointestinal tract. Moreover, the strains exhibited functional characteristics, including substantial antibacterial activity with a minimal inhibitory concentration (MIC) ranging from 12.5 to 50%. They also harbored bacteriocins genes, produced short-chain fatty acids (acetate and propionate), exhibited significant phagocytic activity, possessed high antioxidative properties, rapidly depleted sodium nitrite, and exhibited proteolysis and β-glucosidase activity. In addition, heat-killed LAB strains significantly reduced the gene expressions of proinflammatory cytokines such as IL-β, IL-6, and iNOS in macrophages. Safety assessment revealed no cytotoxicity in macrophage cell lines. All strains tested negative for biogenic amine or H_2_O_2_ production, displayed no gelatinase or hemolytic activity, lacked virulence genes or detrimental enzymes, and displayed antibiotic susceptibility. In summary, these newly isolated strains demonstrate excellent probiotic functionality with a strong focus on safety, making them promising candidates for future drug development in the relevant fields.

**Supplementary Information:**

The online version contains supplementary material available at 10.1007/s00284-024-04034-8.

## Introduction

Lactic acid bacteria (LAB), a group of Gram-positive microorganisms that produce lactic acid as their primary metabolite during fermentation, are distinguished by specific morphological, metabolic, and physiological characteristics [1]. Studies show that LABs can tolerate bile salt and acidic conditions, adhere to intestinal epithelial cells, and function as antimicrobial agents, thus benefitting the host [2]. Among the LAB strains, *Lactobacillus* spp. are widely recognized probiotics that can demonstrate human health benefits through clinical trials [2]. Besides this, Bifidobacterium is also known as an excellent probiotic candidate due to its functional potential [3].

Probiotics are defined as “live microorganisms that can confer a health benefit on the host organism when administered in sufficient amounts,” according to the World Health Organization (WHO) [4]. Probiotics have numerous beneficial properties, including cancer prevention, reduction of blood glucose and cholesterol levels, prevention of obesity and hypertension, and resistance to allergens [2]. Mainly, LAB strains profoundly affect health by stimulating and strengthening the immune system, promoting a healthy gastrointestinal microflora, and regulating the balance of acid and bile salts in the colon [2]. Recently, LAB strains have gained attention for their ability to produce different types of short-chain fatty acids that play a vital role in treating various diseases and disorders [5]. Probiotics have shown astounding success in multidisciplinary sectors, particularly in the biomedical and pharmaceutical industries, demonstrating their importance and therapeutic potential [2].

Probiotics-mediated functional properties have led to advancements in the development of health-enhancing foods and medicines, expanding beyond the traditional focus on fermented foods [6]. Therefore, it is essential to prioritize the cultivation of new strains in order to create outstanding products and maintain competitiveness in this field. Under these circumstances, new strains must be discovered by exploring novel sources distinct from the ones already known. In recent decades, a notable shift has been toward exploring the human microbiome as a potential source for probiotic strains [7]. Hence, this investigation of the human microbiome holds immense potential to identify new and beneficial strains that can significantly contribute to creating innovative probiotic products with heightened functionality and efficacy.

Probiotics are increasingly recognized for their potential in the medical industry due to their wide range of health benefits [8]. However, to effectively develop probiotic strains as a form of treatment, it is necessary to conduct comprehensive functional and safety characteristics studies of the strains in compliance with the regulatory framework. A notable example of such guidelines is the FDA LBP CMC (Live Biotherapeutic Product Chemistry, Manufacturing, and Controls) guideline, which outlines the requirements for developing and regulating probiotic therapies [9]. In our study, we adhered to this guideline, which outlines the requirements for developing and regulating probiotic therapies.

The primary goal of this study was to identify novel probiotic strains from the vaginal fluid of healthy postmenopausal women, which is considered a promising source of probiotics. Subsequently, we conducted various experiments in adherence to rigorous methodologies and guidelines to assess their qualities and potential advantages in depth. Our findings will provide valuable insight into this research area and facilitate a better understanding of probiotic diversity and its therapeutic potential in disease management.

## Materials and Methods

### Selection Criteria of Study Subjects

The research team conducted a vaginal microbiome study using the vaginal fluid collected from healthy postmenopausal women [10]. A postmenopausal woman is defined as a woman who has not menstruated for at least 12 consecutive months [11], and all subjects in this study met this criterion. In addition, the participants did not have any specific medical conditions related to the vagina or the urinary tract, including vaginal dryness, irritation, itchiness, soreness, discomfort while urinating, pain during sexual activity, or bleeding following intercourse. None of the individuals had taken antibiotics or topical estrogen products within a month before the sampling. Informed consent was obtained from each participant before conducting the study. For collecting the vaginal samples, a sterile cotton swab kit was used to collect the vaginal secretion. Then, the cotton swab was immediately placed in 1 ml of sterile saline. All the samples were collected at room temperature and immediately transferred to the PMC laboratory at Soonchunhyang University [10].

### Culturomics-Driven Isolation of LAB Strains from Postmenopausal Vaginal Fluid

A modified culturomics-based method, along with 16S rRNA sequencing, was utilized to isolate LAB strains from healthy postmenopausal vaginal fluid. The vaginal swab samples were freeze-thawed and then diluted up to a 10^–4^ concentration using phosphate-buffered saline (PBS, pH 7.2) (2.7 M NaCl, 54mM KCl, 86 mM Sodium, Phosphate dibasic, 28mM Potassium phosphate monobasic). These diluted samples were cultured on various agar media, including Rogosa SL Agar, MRS agar, and modified MRS agar containing 0.12 g/l of Bromo-cresol purple (Sigma-Aldrich, Germany) and 0.1% of L-cystine HCl (Sigma-Aldrich, Germany). The cultures were incubated at 37 °C for 0–48 h in an anaerobic and microaerophilic chamber (Baker Ruskin, UK). Then, bacterial colonies were selected based on size, shape, color, and morphology and sub-cultured to obtain pure colonies. Plates that did not display any growth initially were further incubated for up to 7 days. Once a single colony of interest was obtained, it was cultured into the respective broth medium and preserved at − 80 °C using a 30% glycerol solution until further use.

### 16S rRNA Sequencing-Based Identification and Phylogenetic Tree Construction of Isolated LAB Strains

16S rRNA sequencing technology was applied in our study to identify probiotic strains at the species level, as it has become a reliable and robust method for identifying and classifying unknown bacterial species. DNA was extracted using the QIAamp DNA Mini Kit (QUIGEN, Germany). The genomic DNA was amplified using the following primers: 27F-AGA GTT TGA TCC TGG CTC AG and 1492R-GGT TAC CTT GTT ACG ACT T. These primer sets were purified and sequenced using the ABI PRISM 3730XL DNA analyzer (Applied Biosystems, USA). Subsequently, the resulting sequencing data was analyzed to identify isolated LAB strains.

Moreover, the obtained sequence was compared to the NCBI (National Center for Biotechnology Information) database using the Basic Local Alignment Search Tool (BLAST) and displayed by constructing a phylogenetic tree on MEGA11 [12]. To construct the dendrogram and calculate evolutionary distances (with bootstrap analysis based on 1000 replications), the Neighbor-Joining method [13] and the Kimura 2-parameter method [14] were employed, respectively.

### Molecular Fingerprinting Analysis of Isolated LAB Strains

A random amplified polymorphic DNA (RAPD) analysis was conducted to distinguish LAB strains from different genera and similar species. The PCR was performed with KAPA HiFi HotStart ReadyMix (KAPA BIOSYSTEMS, South Africa) using the Veriti 96 well Thermal Cycler (Thermo Fisher, USA). The PCR products were separated and visualized using agarose gel electrophoresis and ChemiDoc XRS + gel analyzer (Bio-Rad, USA), respectively. The primer used for this analysis was 5ʹ-AGT CAG CCA C-3ʹ [15].

### Identification of Isolated LAB Strains Based on Carbohydrate Fermentation

Isolated probiotic strains were identified using an API kit (bioMerieux, France) based on carbohydrate fermentation. Initially, LAB strains were sub-cultured under microaerophilic conditions into MRS broth at 37 °C for 24 h. Then, overnight grown LAB strains were washed with 0.85% NaCl; subsequently, a bacterial suspension was prepared by adjusting the turbidity to 2 MacFarland units. Subsequently, 0.1 ml of bacterial suspension was mixed thoroughly into API 50 CHL medium. A tray was then prepared with distilled water, onto which the API 50 CH strip was placed. Finally, mixed bacterial suspension (70 µl) was transferred into the well strip and incubated at 37 °C for 48 h. Following incubation, color changes in each well were observed and recorded (±), and the results were analyzed using APIWEB™.

## Cell Surface Hydrophobicity and Auto-Aggregation

To observe the cell surface hydrophobicity (CSH) of the isolated LAB strains, a previously published method [16] was employed with minor modifications. Overnight-grown LAB strains were washed, and their optical density was adjusted (OD_600_ = 1.0) (A_0_) using a spectrophotometer. Then, *n*-hexadecane (Sigma-Aldrich, Germany) was mixed into the cell suspension (at a ratio of 5:1), vortexed for 2 min, and incubated at 37 °C for 1 h. The aqueous layer was cautiously removed, and the absorbance (A_1_) was measured at OD600 nm. The following formula was used to calculate CSH: CSH (%) = (1–A_1_/A_0_) × 100. To check auto-aggregation (AA), LAB isolates were prepared as described earlier, and the OD was set to 0.6 (A_0_). Then, 5 ml of cell culture mixture was vortexed (10 s) and incubated (1 h), after which the supernatant was removed, and the absorbance (A_1_) was measured; the AA capability was calculated using the previously described formula. *Lactobacillus rhamnosus* (LrGG, KCTC 5033) was used as a reference strain for cell surface hydrophobicity and auto-aggregation.

## Adhesion Assay

Adherence capabilities of isolated LAB strains were determined using an HT-29 cell line obtained from the Korean Cell Line Bank (KCLB 30038, Korea) [17, 18]. HT-29 cell was grown in Dulbecco’s Modified Eagle Medium (DMEM, Gibco, USA) supplemented with 10% (v/v) heat-inactivated fetal bovine serum (FBS, Gibco, USA), and 1% penicillin–streptomycin (Gibco, USA). The cells were incubated at 37 °C in a humidified atmosphere with 5% CO_2_. T-75 cm^2^ flasks (SPL/Corning) were used post-confluence for up to (70 to 90% confluence), with the medium being changed every 2 days. Monolayer cells were detached using 0.05% trypsin and 0.5 mM EDTA (Gibco, USA), washed with PBS, and 1 × 10^5^ cells seeded into 24-well culture plates (SPL/Corning) and incubated under the abovementioned conditions.

Simultaneously, LAB strains were grown, washed, resuspended in PBS (1 × 10^8^ CFU/ml), mixed with fluorescein isothiocyanate (FITC) (100 µg/ml) (Sigma Aldrich, Germany), and incubated at 37 °C for 1 h in the dark. The LAB strains were washed four times with PBS to remove any unlabeled FITC. The monolayer cells were washed 3 times with PBS, and 1 × 10^6^ CFU/ml labeled LAB strains were added to the HT-29 cell. Initial and final fluorescence intensities were measured at 0 h (A_0_) and 2 h (A) using a Victor Nivo multiplate reader, with an excitation wavelength of 485 nm and an emission wavelength of 530 nm. The adhesion was rate calculated as the following equation: A/A_0_ × 100. *L. rhamnosus* (LrGG) was used as a reference strain for adhesion assay.

## Gastric Acid, Bile Salt, and Enzymatic Tolerance of LAB Strains

The survival of isolated LAB strains in simulated gastric acid and bile salt was investigated as previously described [19] with minor modifications. LAB isolates were overnight grown in MRS broth, then centrifuged, washed, and resuspended with PBS. After that, 1% of grown LAB isolate (OD_600nm_ = 0.4) was added into an artificial gastric acid solution, which was prepared by dissolving 0.3% of pepsin (Sigma Aldrich, Germany) in phosphate-buffered saline, and then the pH value was adjusted to 2.5 by adding 1N HCl [19]. Viable bacteria were then enumerated after 0 and 2 h of incubation at 37 °C. To check the bile salt tolerance of grown LAB strains, they were added to MRS broth containing 0.3 and 1% of bile salt (Sigma Aldrich, Germany), and live bacterial numbers were determined at 0 h and 24 h of incubation.

To evaluate the enzyme resistance capability of the test LAB isolates [19], MRS broth was prepared with α-amylase (1 mg/ml, MP Biomedicals, Canada), proteinase K (1 mg/ml, QIAGEN, Germany), trypsin (1 mg/ml, Fisher Scientific, USA), and lysozyme (0.5 mg/ml, Sigma Aldrich, Germany). Then, 0.5% of grown LAB isolates were inoculated into the prepared MRS broth and incubated for 24 h at 37 °C. Then, viable bacteria were enumerated after spreading onto MRS agar. *L. rhamnosus* (LrGG) was used as a reference strain for gastric acid, bile salt, and enzymatic assay.

## Antimicrobial Potentiality of LAB Isolates

The LAB strains’ antimicrobial activities were evaluated using an agar well diffusion test [19] approach against a panel of pathogenic microbes such as *Acinetobacter baumannii* (NCCP 14782), *Clostridium difficile* (ATCC 43255), *Shigella flexneri* (NCCP 14744), *Salmonella typhi* (NCCP 10023), and *Escherichia coli* O157 (NCCP 14541). All the tested pathogenic microbes (except *C. difficile*) were cultured on tryptic soy broth (TSB, Sigma-Aldrich, Germany) incubating at 37 °C overnight. On the other hand, *C. difficile* was cultured on Brain Heart Infusion broth (BHI, Sigma-Aldrich, Germany) and incubated at 37 °C overnight in an anaerobic chamber (Concept 400, Baker Ruskin, UK). 0.5% of the cultured pathogenic strain (OD_600_–0.3) was mixed with nutrient-soft agar and BHI agar (*C. difficile*) (0.7%) (BD, Difco, France) and poured into appropriate agar media. After solidifying the agar, 6-mm wells were created, and 0.1 ml of filtered culture supernatant (pH altered to 6.5) of LAB isolates were spotted in each well and incubated for 18 h at 37 °C aerobically and anaerobically (*C. difficile*). Subsequently, the plates were observed, and clear zones were measured with a vernier caliper to examine the antimicrobial potentiality of LAB isolates.

Antimicrobial activities of our probiotic strains were also explored in terms of minimal inhibitory concentration (MIC) against a panel of vaginal pathogens such as *Prevotella bivia* (KCTC 5454), *Gardnerella vaginalis* (KCTC 5096), *Enterococcus faecium* (LAB isolate), and *Staphylococcus aureus* (LAB isolate; previously isolated from vaginal samples in our lab) and other clinical pathogens (*A. baumannii*, *C. difficile*, *S. flexneri*, *S. typhi*, and *E. coli* O157) [20]. All the pathogens were grown overnight at 37 °C in a BHI broth medium either aerobically (*S. aureus*, *E. faecium*, *A. baumannii*, *S. flexneri*, *S. typhi*, and *E. coli* O157) or anaerobically (*G. vaginalis*, *P. bivia*, and *C. difficile*) and adjusted at 0.5 MacFarland. Subsequently, LAB isolates were grown overnight at 37 °C in MRS broth, centrifuged, transferred the CFS, filtered with a 0.22 µm syringe filter, and stored at 4 °C until used. 100 µl of the diluted CFS (50%, 25%, and 12.5%) from LAB was transferred to 96 well plates and mixed with an equal volume of tested pathogens (MacFarland 0.5). Wells containing 200 µl suspension of pathogens and BHI media were considered positive controls and blanks, respectively. The plates were incubated overnight at 37 °C under aerobic or anaerobic conditions, and MIC was determined by recording the lowest concentration of CFS capable of inhibiting the growth of bacterial pathogens. A broth microdilution turbidimetric assay was also performed to measure bacterial growth inhibition [21]. For the turbidimetric assay, 96 well microplates were prepared by mixing equal volumes of CFS and bacterial pathogens as previously described, and the initial absorbance was measured immediately after mixing at 570 nm using a Victor Nivo multiplate reader. After overnight incubation at 37 °C under aerobic and anaerobic conditions, the final absorbance was measured, and bacterial growth inhibition was calculated, with 100% growth represented by positive control.

## Exploring the pH-Lowering Ability of LAB Strains

The pH-lowering ability of the isolated strain was checked. For this purpose, the strains were grown for 24 h, and their initial pH was checked. Following this, the pH was adjusted to 6.4 using sodium hydroxide (Sigma-Aldrich) and further incubated for 24 h. Finally, pH was checked to verify whether the probiotic culture created the acidic condition.

### Identification of Bacteriocins Encoding Genes

The presence of bacteriocin-producing genes was evaluated using PCR assay. According to the manufacturer’s instructions, genomic DNAs of LAB strains were extracted using the QIAamp DNA Mini Kit (Qiagen, Germany). The PCR was performed using Maxime PCR PreMix (iNtRON Biotechnology Inc., South Korea) master mixer and PCR grade water (Thermo Fisher Scientific, USA) with gene-specific primers. The PCR conditions are provided in Table S9, and the PCR reaction was carried out using the Veriti 96 well Thermal Cycler (Thermo Fisher Scientific, USA). The amplified PCR products were then separated and visualized using agarose gel electrophoresis and ChemiDoc gel analyzer, respectively. The presence of the following genes was examined: antimicrobial genes, including pediocin PA-1 (*pedA1*), nisin (*nisQ*), plantracin (*plan*A*, plan*W, *planS*, and *plan*NC8) [22, 23], and sakicin (*skg*A1, *skg*A2) [24]. Table S9 lists the primer pairs used in this study.

## Determination of Short-Chain Fatty Acid in LAB Strains

The short-chain fatty acid production capabilities of isolated strains were investigated. In the TurboMatrix Headspace, a vial containing 2.5 g of NaCl, 5 ml of the LAB strains (pellet), and 1 ml of 2% sulfuric acid were analyzed. The standard solution underwent the same pretreatment process, with acetic acid, propionic acid, butyric acid, and valeric acid diluted to the concentrations of 1, 2, 5, 10, 20, 50, and 100 mg/l to establish the calibration curve. The analysis was conducted using a gas chromatography-mass spectrometer (GC–MS) with a Clarus 690 coupled with Clarus SQ8 (PerkinElmer) system equipped with a capillary column (Elit-FFAP; 30 m, 0.25 mm i.d, 0.25 µm df; PerkinElmer). The injection port temperature was set to 250 °C, and the injection volume was 0.16 ml/0.08 min with helium (16 ml/min) as the carrier gas in split mode (5:1). The oven temperature was programmed to increase from 120 to 200 °C at the rate of 5 °C per minute. The ion source temperature was adjusted to 250 °C, and the analytes were detected using the full scan mode.

## Phagocytic Activities of LAB Strains

To investigate the phagocytic activities of heat-killed LAB strains in RAW 264.7 cells, the neutral red uptake method [25] was utilized. LAB isolates were cultured overnight at 37 °C in MRS broth, then centrifuged, washed three times, resuspended in PBS, and treated for heat-killing at 100 °C for 30 min. Murine RAW 264.7 cells were grown in Dulbecco’s Modified Eagle Medium (DMEM, Gibco, USA) supplementing 10% fetal bovine serum (FBS) and 1% of penicillin–streptomycin (HyClone, US A) at 37 °C with a 5% CO_2_ humidified incubator. Overnight-grown monolayer cells (2 × 10^5^ cells/well) in a 96 well plate were treated with heat-killed LAB strains (1 × 10^7^ CFU/ml) along with LPS (1 μg/ml) and incubated at 37 °C overnight. Afterward, the cell culture supernatant was replaced with 0.075% neutral red solution and incubated at 37 °C. After 3 h of incubation, cells were washed three times with PBS and lysed with a 50% ethanol, 1% acetic acid, and 49% distilled water solution. The absorbance was then measured at 530 nm using the Victor Nivo multiplate reader to determine the phagocytic activity.

## Free Radical Scavenging Activities of LAB Strains

As previously described, LAB strains’ antioxidant activity was measured using 2,2-diphenyl-1-picrylhydrazyl (DPPH) [26]. LAB strains were grown in MRS broth overnight at 37 °C, centrifuged, transferred the supernatant, and filtered with a 0.22 µm syringe filter. Then, 1 ml of DPPH solution (0.05 mM) was added into 0.1 ml of CFS, vortexed, and incubated for 30 min in the dark at ambient temperature. The absorbances of the mixtures were then measured using a spectrophotometer at 517 nm. The scavenging activity was computed using the following formula: DPPH scavenging activity (%) = 1–(A_s_–A_b_/A_CTRL_) × 100, where A_s_ = absorbance of the DPPH solution with bacterial cell, A_b_ = absorbance of bacterial cells with methanol, and A_CTRL_ = absorbance of DPPH solution only. Again, the scavenging activity of LAB strains was also determined based on a 2,2’-azino-di-(3-ethylbenzthiazoline sulfonic acid) (ABTS) assay as previously described [27]. A mixture of solutions was prepared using an equal ratio of 7 mM ABTS (Sigma-Aldrich, Germany) and 2.45 mM potassium persulfate (Daejung Chemical and Metals, Korea) and incubated overnight at 4 °C in the dark to activate ABTS^+^. Then, 0.3 ml of CFS and 0.6 ml of adjusted OD ABTS^+^ solution (OD_734nm_, 0.7) were mixed. After 30 min of incubation (dark place), the absorbance was measured using a spectrophotometer at 734 nm. The percentage of ABTS scavenging activity was calculated using the following formula described earlier. L-Ascorbic acid (50µM) and PBS were used as standard and control, respectively, for DPPH and ABTS antioxidant analysis.

## Reduction of Sodium Nitrite

Sodium nitrite reduction abilities of the isolated LAB strains were evaluated as previously described [28]. 100 µl of probiotic culture (1 × 10^8^ CFU/ml) was added to the prepared sodium nitrite solution of 150 µg/ml and incubated anaerobically for 12 h at 37 °C. After incubation, they were deproteinated and defatted with ZnSO_4_ (0.42 mol/l) and filtrated using a 0.22 µM filter (GVS Filter Technology, USA). A color-producing solution (0.1% N-1-napthyethylene diamine dihydrochloride, 0.2% sulfanilamide, and 44.5% HCL) was mixed with the filtrate. After incubation at 37 °C in the dark for 5 min, the absorbance was measured at 530 nm using a Victor Nivo multiplate reader (PerkinElmer, USA), and the depletion rate was calculated by using the following equation: Nitrite depletion (%) = (1–A/B) × 100; where A and B was the initial and final amount of nitrite at 0 and 12 h, respectively.

### Proteolytic Activities of Isolated LAB Strains

The potentiality of LAB strains to generate proteolytic enzymes was investigated as previously described [29]. Overnight-grown LAB strains were spotted onto MRS agar plates containing 10% skim milk (MB cells, Korea) and incubated for 24 h at 37 °C. Subsequently, the plates were examined for isolated LAB strains’ proteolytic activity by determining the halos zone against the LAB spots.

## Determination of Whole Cell β-Glucosidase Activity

The *β*-glucosidase activities of isolated LAB strains were determined using 4-nitrophenyl *β*-D-glucopyranoside (PNPG; Sigma-Aldrich, Germany) as a substrate. The bacterial cells were collected via centrifugation from overnight grown cultures and kept at – 20 °C freezer for a certain period. Then, pellets were resuspended using 20 µl of BifiBuffer (1.2 g/l K_2_HPO_4_, 0.333 g/l KH_2_PO_4_, Sigma Alrich, Germany). Then, 2 µl of that resuspended cell suspension was transferred into 198 µl of the PNPG solution (20 mM in BifiBuffer). Then, 100 µl of 1 N Na_2_CO_3_ was added, the solution was incubated for 4 h, and absorbance at 405 nm was measured before and after incubation using a Victor Nivo Multiplate reader.

### Proinflammatory Cytokines and *iNOS* Gene Expression Analysis

The expressions of genes encoding proinflammatory cytokines and *iNOS* were analyzed in RAW 264.7 cells. Overnight-grown monolayer cells were treated with heat-killed LAB strains (1 × 10^7^ CFU/ml) or LPS (1 μg/ml) and incubated at 37 °C for 24 h. Total RNA was then extracted using RNeasy Plus Mini Kit (Qiagen, Germany), and the integrity of total RNA was assessed using agarose gel electrophoresis, and its quantity was measured with a Qubit Fluorometer (Invitrogen, USA) utilizing the Qubit RNA HS Assay Kit (Thermo Fisher, USA). RNA samples were reverse transcribed into cDNA using iScript cDNA Synthesis Kit (Bio-Rad, USA). Real-Time PCR was conducted using SYBR Green Supermix Kit (Bio-Rad, USA) on a CFX96 Real-Time PCR detection system, following the manufacturer’s instructions (Applied Biosystems, USA). The expression levels of the target gene were normalized to the endogenous control gene, glyceraldehyde 3-phosphate dehydrogenase (GAPDH), to investigate the expressions of genes encoding *iNOS* and proinflammatory cytokines. Table S9 lists the primers and PCR conditions used in this study.

## Cytotoxicity Tests of LAB Strains

Cytotoxicity of live and heat-killed LAB strains was determined using RAW 264.7 murine macrophage cell line purchased from Korean Cell Line Bank (KCLB 40071, Korea) [30]. RAW cells were seeded in a 96 well plate at 2 × 10^5^ cells/well and incubated at 37 °C overnight. The Then day, the cells were treated with heat-killed or live LAB strains (1 × 10^8^ (CFU/ml) along with a lipopolysaccharide solution (LPS, 1 µg/ml) (Sigma Aldrich, Germany) as a positive control and incubated at 37 °C overnight. The following day, each well received 20 µl of the WST solution, which was then incubated again for 2 h, and the absorbance was assessed at 570 nm using a Victor Nivo multiplate reader to determine cell cytotoxicity.

### Biogenic Amines, H_2_O_2_, Gelatinase, and Hemolytic Activities of LAB Strains

The production of biogenic amines and gelatinase activity of all LAB strains were explored as previously described [29]. LAB isolates were grown overnight at 37 °C in MRS broth and adjusted to an OD of 1.0 at 600 nm. Then, 0.01% of the LAB strains were added into MRS broth containing 0.1% of individual precursor amino acids (ornithine, tyrosine, histidine, and lysine) (Sigma-Aldrich, Germany) in a ratio of 1:100 and incubated at 37 °C overnight. The following day, a 10 µl culture was spread onto an MRS agar plate containing the same amino acids precursor and incubated at 37 °C overnight. Subsequently, the color change of the media was observed (violet color indicated the presence of biogenic amines) and recorded.

H_2_O_2_ production capacities of LAB strains were investigated following a previously published procedure [31] with some modifications. They were streaked onto MRS agar containing 0.25 mg/ml of 3, 3ʹ, 5, 5ʹ-tetramethylbenzidine (Sigma-Aldrich, Germany) and 0.01 mg/ml of horseradish peroxidase (Sigma-Aldrich, Germany) and incubated anaerobically at 37 °C. After 48 h of incubation, the plates were placed in the atmosphere for a certain period to observe a blue color change, indicating H_2_O_2_ production of the test strains.

To evaluate gelatinase activity, 10 µl of LAB culture was inoculated into Luria Bertani broth (BD, USA) containing 2% agar and 3% gelatin (Duksanpure Chemicals Co., Korea) and incubated at 37 °C for 4 days. Then, the gelatin hydrolysis activities of LAB strains were observed after keeping them at 4 °C for 4 h. The hemolytic activities of the isolated LAB isolates were also investigated. LAB strains were cultured in Tryptic Soy Agar (Merk, Germany) supplemented with 5% sheep blood defibrinated (MB cells, Korea) and incubated at 37 °C for 48 h. Then, clear zones encircling bacterial culture were observed and interpreted as signs of complete hemolysis (*α*), partial hemolysis (*β*), or no hemolysis (*γ*).

### Detection of Virulence Genes, Vancomycin Resistance Genes, and Biogenic Amine Genes

According to the manufacturer’s instructions, genomic DNAs of LAB isolates were extracted from overnight growing LAB cultures using QIAamp DNA Mini Kit (QIAGEN, Germany). DNA integrity was checked using agarose gel electrophoresis and quantified by a Qubit Fluorometer (Invitrogen, USA) utilizing the Qubit DNA HS Assay Kit (Thermo Fisher, USA). Conventional PCR was performed to observe the presence of virulence genes, vancomycin resistance genes, and biogenic amine genes using Maxime PCR PreMix (iNtRON Biotechnology Inc., South Korea) master mixer and PCR grade water (Thermo Fisher Scientific, USA) with gene-specific primers. In this study, the presence of the following genes was examined: adhesion gene *ace* (adhesion of collagen), aggregation gene *asa1* (aggregation substance), virulence genes including *cylA* (cytolysin), *esp* (enterococcal surface protein), *gelE* (gelatinase), *hyl* (hyaluronidase), and *efaA* (endocarditis antigen), vancomycin resistance genes *vanA* and *vanB*, and biogenic amines *hdc* (histidine decarboxylase), *odc* (ornithine decarboxylase), and *tdc* (tyrosine decarboxylase) [22, 32]. Primer pairs and PCR conditions used in this study are listed in Table S9.

## Enzymatic Activities of LAB Strains

An API ZYM kit (BioMerieux, France) was used to explore the enzyme-producing capacity of isolated LAB strains. Cultured LAB strains were adjusted to 5 McFarland, and a 75 µl cell suspension was aliquoted into each strip well and incubated for 6 h at 37 °C. After that, each cupule was filled with drops of ZYM A and ZYM B reagents and further incubated at an ambient temperature for 10 min. The enzymatic activities were then categorized based on the intensity of color change (0–5; 0 indicating no activity and 5 indicating the maximum activity).

## Antibiotic Susceptibility Assay of LAB Strains

Antibiotic susceptibilities of LAB isolates were determined using the agar disk diffusion method [33]. For this study, the following commercial antibiotics were purchased and used: carbenicillin (CAR, 100 µg), cefoxitin (FOX, 10 µg), clindamycin (CD, 2 µg), chloramphenicol (C, 30 µg), erythromycin (E, 15 µg), metronidazole (MZT, 5 µg), ampicillin (AMP, 10 µg), tetracycline (TE, 30 µg) (from Liofilchem®, Italy), streptomycin (S, 30 µg) (from BD BBL™, Sensi-Disk, USA), ciprofloxacin (CIP, 5 µg) and amikacin (AK 30 µg) (from Oxoid™, USA). Precultured LAB isolates were adjusted to 0.5 McFarland. Bacterial lawns were then prepared on MRS agar plates. Then, antibiotic discs were manually placed onto the surfaces of bacterial lawns and incubated at 37 °C overnight. The resulting zone of inhibition was measured in diameter, and antibiotic susceptibility was categorized as S, Sensitive (≥ 17.5 mm); IR, Intermediately resistant (12.5–17.4 mm); or R, Resistant (≥ 12.4 mm) [34].

An E-test was conducted to determine the antibiotic susceptibility of the candidate LAB strain, following the guidelines of CLSI, and then determined the MIC recommended by the European Food Safety Authority (EFSA) [35]. A bacterial inoculum was prepared (0.5 McFarland) and subsequently inoculated onto the Mueller–Hinton agar plate. Following this, E-test strips (BioMerieux, France), impregnated with a gradient of various antibiotics (ampicillin, gentamicin, kanamycin, streptomycin, erythromycin, clindamycin, tetracycline, and chloramphenicol), were then placed on the agar plates and incubated for 2 days at 37 ℃ under anaerobic conditions. The minimum inhibitory concentration (MIC), the lowest antibiotic concentration that completely inhibited bacterial growth, was subsequently determined and compared to ESFA’s MIC cut-off criteria.

### Statistical Analysis

All experiments were performed in triplicate. All data are presented as mean ± standard deviation (SD). Statistical significance was determined using one-way ANOVA, Dunnett (GraphPad Prism, version 8.0.1). The significance level was defined as *P* < 0.05. Here, it is noteworthy to mention that after collecting the data, we checked the normality of the data based on histogram techniques.

### Accession Number of Nucleotides Sequence

The 16S rRNA gene nucleotide sequences identified in this study have been deposited to the GenBank database with accession numbers PQ345789-PQ345796 (Table S2).

## Results

### Isolation and Identification of Isolated LAB Strains

An optimized culturomics-based technique used to isolate LABs strain from postmenopausal vaginal fluid is illustrated schematically in Fig. S1. A total of 45 vaginal samples were collected for analysis, and among them, 11 samples exhibited bacterial growth. We selected 69 bacterial colonies from these samples based on the observed color changes and morphology (Table S1). Among the selected colonies, 8 isolates were identified as LAB strains (Table S2), resulting in their essential characteristics and functional properties being explored. In contrast, other isolated strains, apart from *E. faecium* and *S. aureus* (Table S10), which were used to evaluate the antimicrobial effect of our probiotic strain, were excluded from the study (Table S3).

Single pure colonies were selected and sent for 16S rRNA gene sequencing to confirm further and validate their taxonomy. Among them, eight LAB strains were obtained, which belonged to five species on chromas sequence analysis and NCBI BLAST analysis (99–100% identities, Table S2). These eight LAB strains included: three isolates of *Lactiplantibacillus plantarum* (LP1, LP5, LP6), two isolates of *Pediococcus acidilactici* (PA2, PA7), one isolate of *Limosilactobacillus fermentum* (LF3), one isolate of *Lacticaseibacillus paracasei* (LPa4), and one isolate of *Latilactobacillus sakei* (LS8).

**Fig. 1:**
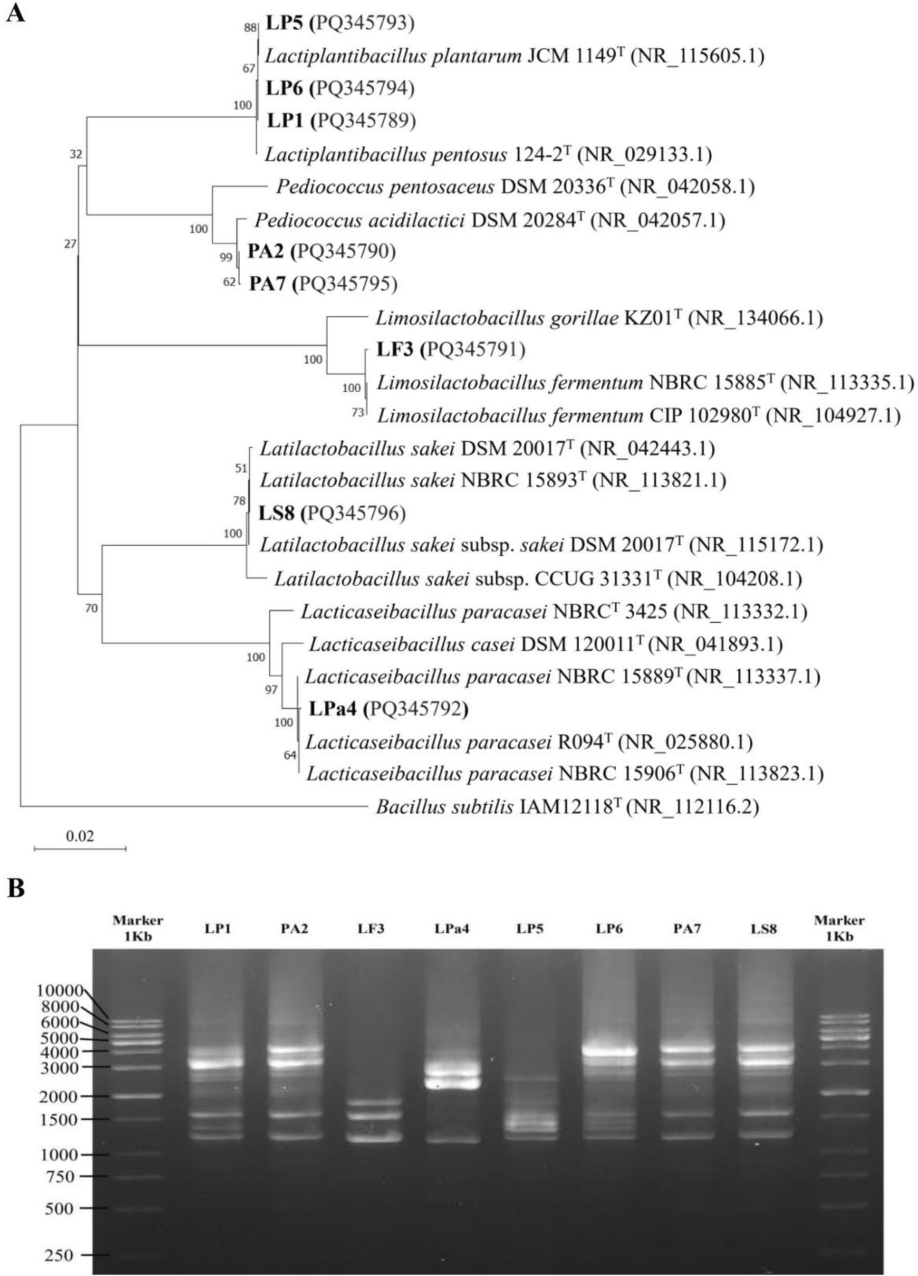
Phylogenetic tree construction and molecular fingerprinting analysis. **a** Using the Neighbor-Joining method (MEGA 11), the phylogenetic tree was constructed based on 16S rRNA sequence homology, revealing five distinct groups containing eight LAB strains. Specifically, LP1, LP5, and LP6 clustered together with *Lactiplantibacillus plantarum*; PA2 and PA7 grouped with *Pediococcus acidilactici*; and LF3, Lpa4, and LS8 clustered with *Limosilactobacillus fermentum, Lacticaseibacillus paracasei,* and *Latilactobacillus sakei,* respectively. *Bacillus subtilis* was used as an outgroup. **b** Molecular fingerprinting analysis based on the Randomly Amplified Polymorphic DNA (RAPD) technique revealed the genetic pattern of isolated LAB strains, wherein 3 to 9 different bands were identified, indicating the distinctiveness of individual strains

Moreover, the phylogenetic tree analysis revealed that LP1, LP5, and LP6 isolates are grouped with *Lactiplantibacillus plantarum* (Fig. 1a). PA2 and PA7 isolates are clustered with *Pediococcus acidilactici*, while LF3, LPa4, and LS8 isolates clustered with *Limosilactobacillus fermentum*, *Latilactobacillus sakei*, and *Lacticaseibacillus paracasei*, respectively. The RAPD typing tool was used to identify individual polymorphism among the tested LAB strains (Fig. 1b). The RAPD marker successfully amplified all the isolates, resulting in 3–9 bands, indicating their genetic similarity and dissimilarity.

Furthermore, the carbohydrate-based identification approach identified three isolates (i.e., LP1, LP5, and LP6) as *L. plantarum*, one (LF3) as *L. fermentum,* and another one (LPa4) as *L. paracasei* ssp. *paracasei* 1, wherein the similarity rate was 99.9%, 99.2%, and 99.9%, respectively (Table S4). Similarly, two isolates (PA2 and PA7) were identified as *Pediococcus pentosaceus* 2, showing 98.2% similarities, but one isolate (LS8) was identified as *Leuconostoc mesenteroides spp. mesenteriodes* (99.9%).

### Exploring Cell Surface Hydrophobicity, Auto-Aggregation, and Adhesion Capabilities of LAB Isolates

As determined on MRS-BP agar, all the tested LAB isolates were acid producers (Table 1). Cell surface hydrophobicity and auto-aggregation of the isolated LAB strains are shown in Table 1. All tested isolates displayed hydrophobicity above 76%. Among them, LF3, LPa4, LP5, LP6, and LrGG isolates showed hydrophobicity of 95.74%, 94.16%, 95.98%, 97.38%, and 97.2%, respectively, which is similar to the control strain. All tested LAB isolates exhibited auto-aggregation of 17–61%. Among these strains, LP5 displayed the highest (61.06%) auto-aggregation compared to the control strain LrGG (21%).

**Table 1:** Acid production, cell surface hydrophobicity, auto-aggregation, and adhesion capability of isolated lactic acid bacterial strains

Isolates	Acid production (MRS + BP)	Cell surface hydrophobicity (CSH %)	Auto-aggregation (AA %)	Adhesion rate (%)
LP1	+	82.28 ± 0.03	29.68 ± 1.37	21.3 ± 0.06
LP5	+	95.98 ± 0.03	61.06 ± 0.10	9.7 ± 0.37
LP6	+	97.38 ± 0.01	30.99 ± 0.06	8.7 ± 0.38
LF3	+	95.74 ± 0.01	34.85 ± 0.63	17.8 ± 0.24
LPa4	+	94.16 ± 0.05	37.97 ± 0.22	12.2 ± 0.35
PA2	+	76.03 ± 0.30	17.77 ± 0.28	21.8 ± 0.33
PA7	+	83.72 ± 0.00	17.46 ± 0.18	23.6 ± 0.13
LS8	+	86.49 ± 0.17	22.01 ± 0.38	34.3 ± 0.4
LrGG	+	97.2 ± 0.14	20.68 ± 0.25	37 ± 0.34

MRS, De man, Rogosa, and Sharpe agar; BP, bromocresol purple; CSH, cell surface hydrophobicity; AA, auto-aggregation. ‘ + ’ indicates acid production. LP1, LP5, and LP6 belong to *Lactiplantibacillus plantarum*; PA2, and PA7 belong to *Pediococcus acidilactici*; LF3, LPa4, LS8, and LrGG belong to *Limosilactobacillus fermentum*, *Lacticaseibacillus paracasei*, *Latilactobacillus sakeii*, and *Lactobacillus rhamnosus*, respectively Values are presented as mean ± SD of triplicate measurements. *ND* Not determined

The adherence abilities of LAB strains for HT-29 cells are shown in Table 1. Among the LAB isolates, LP1, PA2, LF3, PA7, LS8, and LrGG displayed high adhesion abilities of 20.75%, 22%, 18.05%, 24.1%, 34.15%, and 37.34%, respectively, which is consistent with the control strain LrGG. On the other hand, LPa4, LP5, and LP6 showed low adhesion abilities of 12%, 10%, and 8.75%, respectively.

### Evaluating the Tolerance Ability of LAB Strains in Gastric Acid, Bile Salts, and Digestive Enzymes

**Fig. 2:**
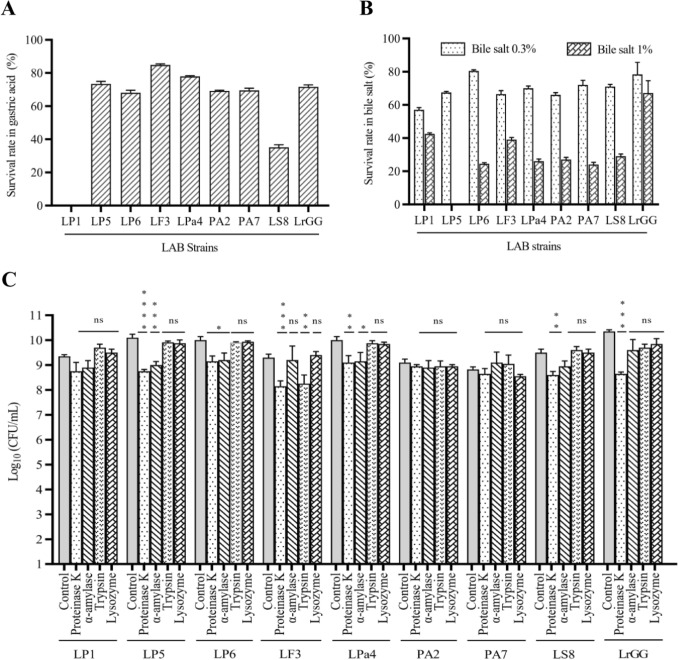
The tolerance of LAB strains to gastric acid, bile salt, and digestive enzymes. The survivability of LAB strains in artificial gastric acid, ox gall bile salt, and digestive enzymes was evaluated. **a** Most lactic acid bacterial (LAB) strains, except for LP1, resisted artificial gastric acid in the acid tolerance assay. **b** Similarly, when the LAB strains were subjected to bile salt, robust tolerance was noted at 0.3% bile salt concentration. **c** LAB strains also displayed excellent survivability in different digestive enzymes compared to the control group. The experiment was conducted in triplicate, and the results are presented as the mean ± SD. Statical significances (*, *p* < 0.05; **, *p* < 0.01; ***, *p* < 0.001; ****, *p* < 0.0001) were determined using one-way ANOVA with Graph Pad Prism software (version 8.0.1)

The survivability of LAB strains against gastric juice, bile salt, and digestive enzymes was depicted in Fig. 2. The results indicated that all LAB strains, except LP1, could survive at least 2 h in acidic conditions (pH 2.5). Among all the LAB isolates, the LF3 strain displayed the highest survival rate of 84% compared to the control strain LrGG at 75%. In comparison, the others showed survival rates of above 67%, except for LS8, which had a survival rate of 34% (Fig. 2a). The survivability of LAB strain against 0.3 and 1% of bile salt concentration ranged from 58 to 81% and 0 to 43%, respectively. Meanwhile, the control strain LrGG showed survivability rates of 78% at 0.3% and 67% at 1%. LP1 isolates, sensitive to low acidic conditions, were well-tolerated (43%) in 1% of bile salt (Fig. 2b).

The LAB isolates’ tolerance to the digestive enzymes was evaluated and shown in Fig. 2C. The results indicated that most isolates survived in the presence of these enzymes. We did not notice any significant difference in survivability between the LAB isolates LP1, PA2, and PA7 and the control group, indicating the enzyme tolerance capability of these strains. However, the growth of LP5 was significantly decreased in the presence of proteinase K (*P* < 0.0001) and *α*-amylase (*P* < 0.001), and the growth of LF3 isolate was significantly decreased in the presence of proteinase K (*P* < 0.001) and trypsin (*P* < 0.01). On the other hand, control strain LrGG also showed a significant decrease only in proteinase K (*P* < 0.001), consistent with other LAB strains.

### Evaluation of Antimicrobial Activity, pH-Lowering Ability, Bacteriocin Genes Presence, Short-Chain Fatty Acids, and Phagocytosis Capability

The antimicrobial properties of the tested LAB strains were studied (Table 2). All LAB strains displayed a clear zone of inhibition (mm) against *Acinetobacter baumannii, Clostridium difficile, Shigella flexneri,* and *Salmonella typhi* but not against *E. coli* O157 (except for isolates LP5 and LP6).

**Table 2:** Evaluating the antimicrobial activity of lactic acid bacterial strains against pathogenic microorganisms

Isolates	Indicator pathogens				
	*Acinetobacter baumannii*	*Clostridium difficile*	*Shigella flexneri*	*Salmonella typhi*	*Escherichia coli O157*
LP1	8.67 ± 0.76	10.17 ± 0.29	14.17 ± 0.76	10.33 ± 0.76	-
LP5	10.50 ± 0.50	15.50 ± 0.50	13. 17 ± 0.76	10.50 ± 0.50	9.17 ± 0.29
LP6	11.33 ± 0.29	16.33 ± 0.76	15.17 ± 0.76	11.83 ± 0.76	9.17 ± 0.29
LF3	8.17 ± 0.29	10.17 ± 0.76	12.50 ± 0.50	-	-
LPa4	6.83 ± 0.29	12.17 ± 0.29	11.67 ± 0.29	7.00 ± 0.50	-
PA2	10.67 ± 0.76	14.67 ± 0.29	14.50 ± 0.50	9.83 ± 0.76	-
PA7	15.00 ± 0.50	13.83 ± 0.29	12.00 ± 0.50	11.17 ± 0.76	-
LS8	13.67 ± 0.76	12.83 ± 0.76	14.17 ± 0.76	6.50 ± 0.50	-

Antimicrobial activity was determined in terms of the inhibition zones expressed in millimeters; ‘-’ indicates no zone of inhibition. LP1, LP5, and LP6 belong to *Lactiplantibacillus plantarum*; PA2 and PA7 belong to *Pediococcus acidilactici*; LF3, LPa4, and LS8 belong to *Limosilactobacillus fermentum*, *Lacticaseibacillus paracasei*, and *Latilactobacillus sakeii*, respectively. Here, values are presented as mean ± SD of triplicates (mm)

Using the broth microdilution assay, the lowest concentration of MICs was determined as 12.5% CFS (LP1, LPa4, LP5, LP6, and PA7) and 25% CFS (PA2, LF3, and LS8) against *P. bivia* (Table 3). MICs at 25% CFS were also found for LP1, LP5, and LP6 against *S. aureus*, *A. baumannii*, and *S. flexneri*, respectively. Furthermore, *G. vaginalis*, *E. faecium*, *A. baumannii*, *C. difficile*, *S. typhi*, and *E. coli O157* were inhibited by 50% CFS from all LAB isolates (Table 3).

**Table 3:** Determining the minimal inhibitory concentration (MIC) of cell-free supernatant of LAB isolates against pathogenic microbes

Cell-free supernatant (%)	Tested pathogens								
	*Prevotella bivia*	*Gardnerella vaginalis*	*Staphylococcus aureus*	*Enterococcus faecium*	*Acinetobacter baumannii*	*Clostridium difficile*	*Shigella flexneri*	*Salmonella typhi*	*Escherichia coli O157*
LP1	12.5%	50%	25%	50%	25%	50%	25%	50%	50%
LP5	12.5%	50%	50%	50%	25%	50%	25%	50%	50%
LP6	12.5%	50%	50%	50%	25%	50%	25%	50%	50%
LF3	25%	50%	50%	50%	50%	50%	50%	50%	50%
LPa4	12.5%	50%	25%	50%	50%	50%	25%	50%	50%
PA2	25%	50%	25%	50%	50%	50%	50%	50%	50%
PA7	12.5%	50%	25%	50%	50%	50%	50%	50%	50%
LS8	25%	50%	50%	50%	50%	50%	50%	50%	50%

The Minimal inhibitory concentration of cell-free supernatant (CFS) from LAB isolates was determined using a broth microdilution assay against a panel of pathogens, including vaginal pathogens. The CFS concentrations of 50%, 25%, and 12.5% were chosen to assess their inhibitory effects on the pathogenic microbes. The isolates LP1, LP5, and LP6 belong to *Lactiplantibacillus plantarum*; PA2 and PA7 belong to *Pediococcus acidilactici*; LF3, LPa4, and LS8 belong to *Limosilactobacillus fermentum*, *Lacticaseibacillus paracasei*, and *Latilactobacillus sakeii*, respectively

The turbidimetric microtiter plate assay result revealed diversified results for the CFS activities of the LAB isolates against the vaginal and clinical pathogens (Fig. S2). Among the tested pathogens, *P. bivia* and *S. aureus* (except LS8) were inhibited by ≥ 98%; *A. baumannii*, *C. difficile* (except LS8), and *S. flexneri* (except LF3, PA2, PA7, and LS8) were inhibited by ≥ 90% using 50% CFS co-cultured with the pathogens. Additionally, 90% growth inhibition was observed for *G. vaginalis* (LP1 and LF3) and *E. faecium* (LP5 and LP6) using 50% CFS of LAB isolates. In contrast, *E. faecium* (except LP5 and LP6, 12–95%), *S. typhi* (21%−32%), and *E. coli* O157 (28–35%) showed higher growth rates when treated with 50% of CFS.

The ability of the isolated strains to lower the pH was evaluated (Fig. S3). Data showed the initial pH of the strain, LP1, Lp5, Lp6, LF3, LPa8, PA2, PA7, and LS8 after 24 h of incubation was 3.91.3.94, 3.96, 4.01, 4.11, 4.22, 3.88, and 4.32, respectively. Following an additional 24 h of incubation, the pH was lowered slightly. On the other hand, in the pH-adjusted group, a significant pH reduction was observed at 48 h of incubation from adjusted pH 6.4 to 3.92.3.93, 3.95, 4.04, 4.21, 4.25, 3.88, and 4.22, respectively.

The production capabilities of bacteriocins in the LAB strains were also assessed, and the findings are presented in Table 4. LAB isolates, LP1, PA2, LP6, PA7, and LS8, showed positive results for pediocin PA-1, while other isolates showed negative results for its presence. In the case of *plan*S and *plan*A, isolates LP1, PL5, and LP6 showed positive outcomes. None of the tested isolates displayed positive results for the *nis*Q, *plan*W, *plan*NC8, *skg*A1, or *skg*A2 bacteriocin gene.

**Table 4:** Determining the presence of bactericidal genes in the isolated lactic acid bacterial strains

Target genes		LAB Strains							
		LP1	LP5	LP6	LF3	LPa4	PA2	PA7	LS8
Antimicrobial genes	*ped* (Pediocin PA-1)	+	–	+	–	–	+	+	+
	*nis*Q (Nisin)	–	–	–	–	–	–	–	–
	*plan*W (Plantaracin W)	–	–	–	–	–	–	–	–
	*plan*S (Plantaracin S)	+	+	+	–	–	–	–	–
	*plan*NC8 (Plantaracin NC8)	–	–	–	–	–	–	–	–
	*plan*A (plantaracin A)	+	+	+	–	–	–	–	–
	*skg*A1 (Sakacin)	–	–	–	–	–	–	–	–
	*skg*A2 (Sakacin)	–	–	–	–	–	–	–	–

‘ + ’ indicates the presence of the targeted genes while ‘–’ indicates its absence

**Fig. 3:**
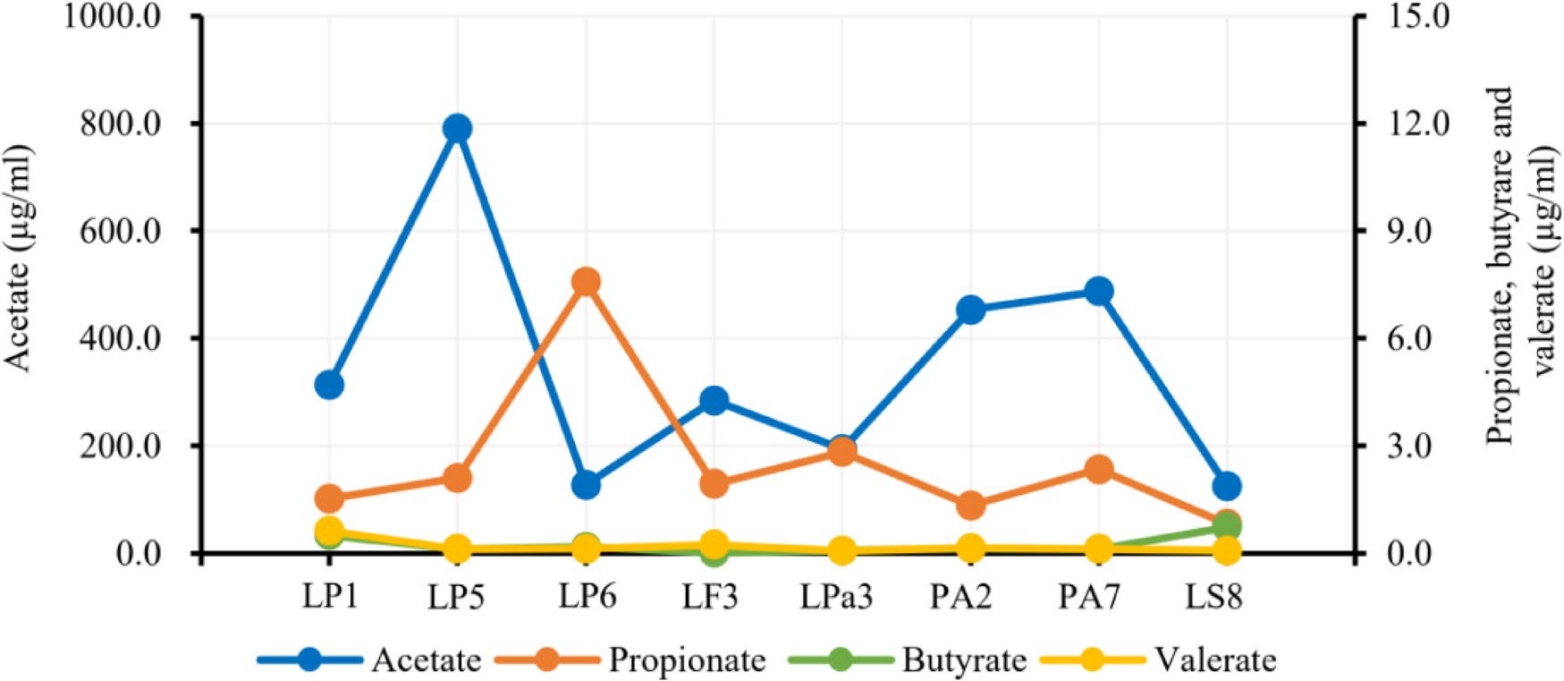
Production of short-chain fatty acids (SCFAs) by lactic acid bacteria (LAB) strains. Short-chain fatty acid (SCFA) production capability in LAB strains was evaluated. The gas chromatography–mass spectrometry assay demonstrated the significant production of acetic acid (blue line; highest value, 790.54 µg/ml) and propionic acid (orange line; highest value, 7.57 µg/ml), whereas butyric acid (green line; highest value, 0.77 µg/ml) and valeric acid (yellow line; highest value, 0.63 µg/ml) was produced in trace quantities

Short-chain fatty acids (SCFAs) analysis revealed that all LAB strains produced a variety of different concentrations of SCFAs, including acetate, propionate, butyrate, and valerate (Fig. 3). Overnight-grown bacterial pellet demonstrated acetate (blue line) producing ability of 127.24–790.54 µg/ml. Among the isolates, LP5 showed the highest acetate production (790.54 µg/ml), whereas LP6 showed the lowest (127.24 µg/ml) production. The LAB isolates produced propionate (orange line) ranging from 0.81 to 7.57 µg/ml, wherein isolate LP6 displayed the highest production. The probiotic strain also produced butyric acid (green line) and valeric acid (yellow line) in the range of 0.03–0.77 µg/ml and 0.08–0.63 µg/ml, respectively**.**

**Fig. 4:**
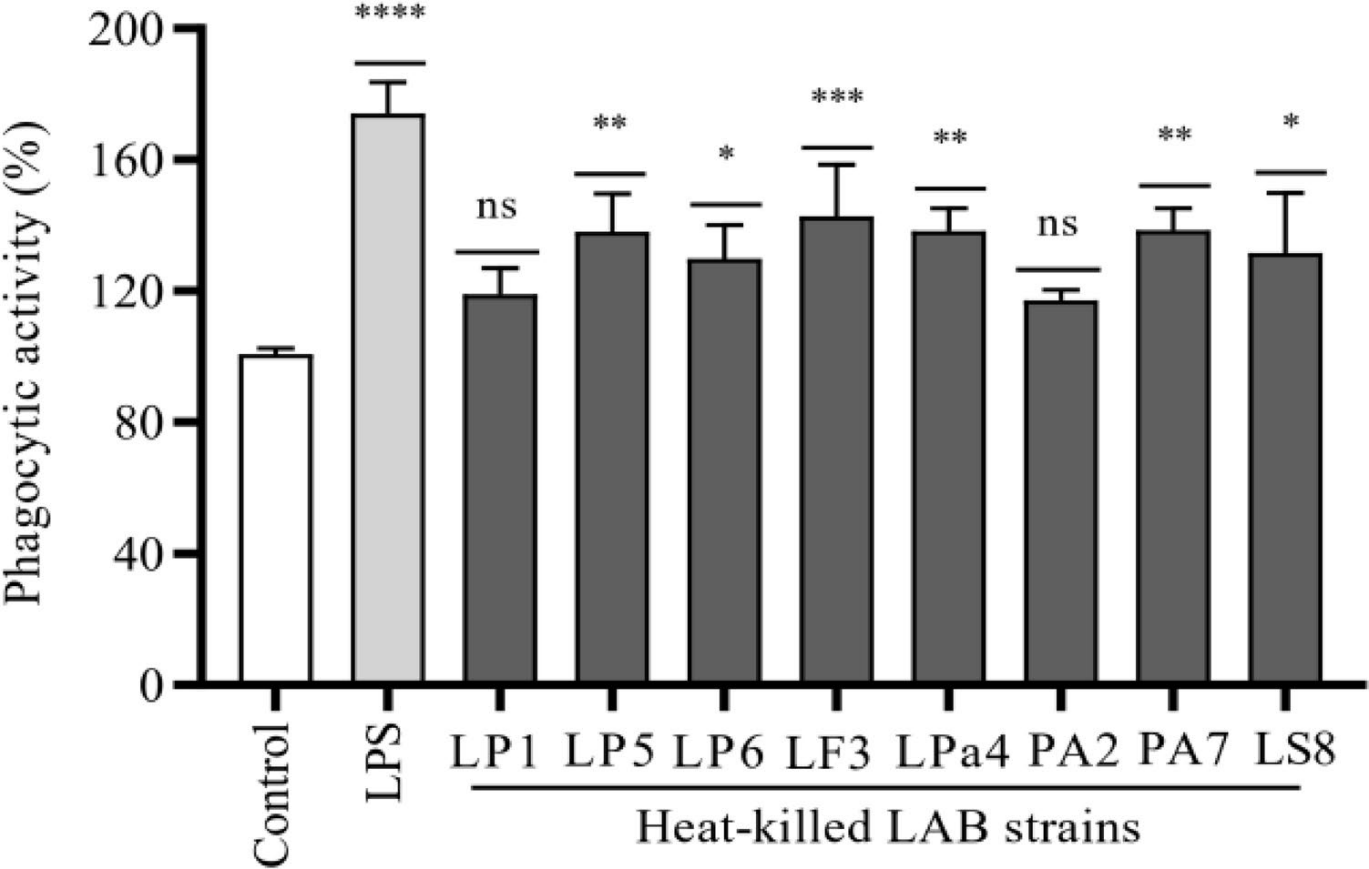
Phagocytic activity of heat-killed LAB strains on RAW 264.7 cells. The phagocytic activity of heat-killed LAB strains (1 × 10^7^ CFU/ml) was evaluated using macrophage cells. The neutral red uptake method significantly elevated phagocytic activity in cells treated with heat-killed LAB strains or LPS compared to untreated cells. The data presented as the mean ± standard deviation derived from triplicate experiments. Statistical significance (*, *p* < 0.05; **, *p* < 0.01; ***, *p* < 0.001; and ****, *p* < 0.0001) was determined using one-way ANOVA (Graph Pad Prism, version 8.0.2)

The effect of heat-killed LAB strains on the phagocytic activity of RAW 264.7 cells was determined and shown in Fig. 4. The results revealed that all LAB strains except LP1 and PA2 increased phagocytic activity significantly compared to the control group. Among the tested LAB strains, LF3 increased the phagocytic activity the most (*P* < 0.001), followed by LP5, LPa4, and PA7 (*P* < 0.01), and LP6 and LS8 (*P *< 0.05).

### Determining the Antioxidant Activity, Sodium Nitrite Depletion, Proteolysis, and *β*-Glucosidase Potentiality of Isolated LAB Isolates

The antioxidant activity of LAB strains was determined, and their free radical scavenging activities for DPPH and ABTS are displayed in Table 5. All LAB isolates displayed a high DPPH scavenging ability of 34 to 78%. Among these isolates, PA2 showed the highest free radical scavenging ability at 78%, compared to ascorbic acid at 99%. In contrast, LPa4 showed the lowest scavenging ability at 34%. LAB isolates’ ABTS free radical antioxidant activity ranged from 44 to 82%. PA2 showed the highest antioxidant activity at 83%, while LPa4 showed the lowest antioxidant ability at 44%, compared to ascorbic acid at 98%.

**Table 5:** Antioxidative activity, nitrate depletion capacity, proteolysis, and β-glucosidase activity of the isolated lactic acid bacterial strains

Isolates	DPPH scavenging activity (%)	ABTS scavenging activity (%)	Depletion of sodium nitrite (%)	Proteolysis	β-glucosidase activity
LP1	62.12 ± 1.57	82.38 ± 0.32	83.03 ± 0.16	+	–
LP5	47.29 ± 1.20	61.57 ± 2.26	91.00 ± 0.19	+	+ ^a^
LP6	52.18 ± 0.59	78.62 ± 0.21	89.31 ± 0.04	+	–
LF3	65.28 ± 1.67	74.10 ± 0.22	72.24 ± 0.79	+	+
LPa4	34.15 ± 0.78	44.14 ± 1.26	79.08 ± 0.81	+	+ ^a^
PA2	78.45 ± 1.57	83.10 ± 0.16	77.81 ± 0.72	+	–
PA7	69.78 ± 0.39	80.12 ± 0.14	87.99 ± 0.48	+	–
LS8	72.39 ± 1.25	81.00 ± 0.13	90.27 ± 0.08	+	–
Ascorbic acid	98.95 ± 0.21	97.87 ± 0.89	ND	ND	ND

DPPH, 2,2-diphenyl-1-picrylhydrazyl; ABTS, 2,2’-azino-di-(3-ethylbenzthiazoline sulfonic acid). Here, “ + ”, positive; “–”, negative; “ + ^a”^, strong activity. Here, values are presented as mean ± SD of triplicate analysis. *ND* Not determined

The results of sodium nitrite depletion and proteolysis ability of LAB isolates are shown in Table 5. Our study data revealed that the isolated LAB strains could reduce nitrite levels by 72 to 91% within 12 h. Among these isolates, LP5 and LS8 had the highest nitrite depletion rates of 91 and 90%, respectively, whereas LF3 showed the lowest nitrite depletion rate of 72%. In addition, all LAB isolates showed the ability to hydrolyze milk protein by appearing as a halo surrounding the LAB spots, suggesting their proteolytic properties.

The *β*-glucosidase-producing capacities of LAB strains were evaluated using *β*-glucosidase assay (Table 5). Whole-cell *β*-glucosidase activities of LAB isolates were also determined using PNPG as the substrate. Only the isolated LP5, LF3, and LPa4 demonstrated positive outcomes. LP5 and LPa4 showed substantial *β*-glucosidase activities, while the other LAB isolates tested negative for *β*-glucosidase.

### Gene Expression Analysis of Proinflammatory Cytokines and *iNOS*

**Fig. 5:**
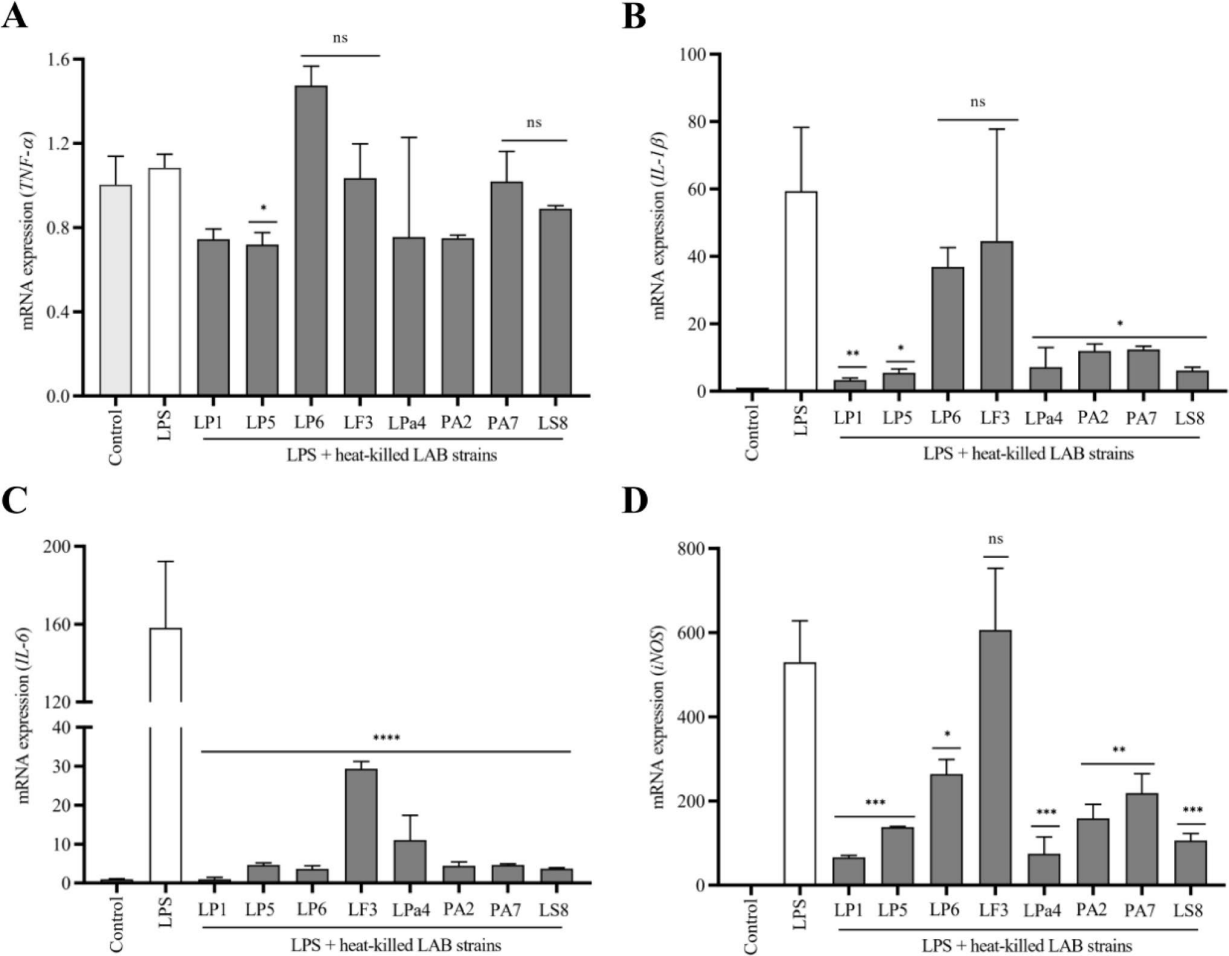
Immunomodulatory effect of heat-killed LAB strains on macrophage cells. The immunomodulatory impact of heat-killed LAB strains (2.5 × 10.^7^ CFU/ml) regarding mRNA expression was assessed. RT-PCR assay showed the effect of LAB strains in downregulating the **a** tumor necrosis factor-α (*TNF-α*) gene, **b** interleukin-1β (*IL-1β*), **c** interleukin-6 (*IL-6*), and **d** *iNOS* genes relative to the control. The results are presented as the mean ± standard deviation of triplicate experiments. Statistical significance was determined by comparing the obtained data to the untreated group using Graph Pad Prism version 8.0.1 with one-way ANOVA (*, *p* < 0.05; **, *p* < 0.01; ***, *p* < 0.001; and ****, *p* < 0.0001)

The immunomodulatory effects of heat-killed LAB strains revealed that the gene expressions of *TNF-α*, *IL-1β*, *IL-6*, and *iNOS* were increased by treatment with LPS but significantly decreased after treatment with heat-killed LAB strains (Fig. 5). In the case of *TNF-α*, its gene expression was notably reduced after treatment with LP5 but not significantly decreased after treatment with other LAB isolates (Fig. 5a). In the case of *IL-1β*, most isolates reduced their gene expression significantly except LP6 or LF3 (Fig. 5b). The *IL-6* expression was significantly reduced after treatment with all LAB strains (Fig. 5c). The *iNOS* expression was also significantly decreased after treatment with all LAB isolates except LF3 (Fig. 5d).

## Assessing the Cytotoxicity of LAB Strains

Cytotoxicity of heat-killed and live LAB isolates was evaluated using RAW 264.7 cells and illustrated in Fig. S4. No cytotoxicity (cell viability ≥ 108%) was observed at a concentration of 1 × 10^8^ CFU/ml for heat-killed LAB strains. In contrast, all RAW 264.7 cells treated with live LAB strains (except LF3) showed lower cell viability (≤ 102%) compared to RAW 264.7 cells treated with heat-killed LAB strains. Among the LAB strains tested, LPa4 exhibited the highest level of cell cytotoxicity at 51%.

### Evaluating Biogenic Amines, H_2_O_2_, Hemolysis, and Gelatinase Activities

The production of biogenic amines, H_2_O_2_, hemolysis, and gelatinase activities of LAB strains are presented in Table S5. The study showed that none of these LAB isolates produced H_2_O_2_ or other biogenic amines, including histamine, ornithine, lysine, and tyrosine. In addition, they showed no *α* (complete hemolysis) or *β*-hemolytic (partial hemolysis) activities on the blood agar medium, indicating that they were all *γ*-hemolytic (no hemolysis). Moreover, all tested LAB isolates were gelatinase-negative.

### Determination of Virulence Genes, Vancomycin Resistance Genes, and Biogenic Amines Genes

The occurrence of virulence genes, genes for vancomycin resistance, and biogenic amines revealed that none of the tested LAB isolates had virulence genes (*gel*E, *hyl*, *asa*1, *esp*, *cyl*A, *efa*A, and *ace*), vancomycin resistance genes (*van*A and *van*B), and biogenic amines producing genes (*hdc*A, *tdc*A, and *odc*A), indicating the safety properties of these strains (Table S6).

### Assessment of Enzymatic Activities of Isolated LAB Strains

The enzymatic activities of the tested LAB strains are shown in Table S7. None of these tested LAB strains produced alkaline phosphatase, lipase, trypsin, *α*-chymotrypsin, *β*-glucuronidase, *α*-mannosidase, or *α*-fucosidase. However, esterase, *α*-galactosidase, *β*-galactosidase, and *β*-glucosidase activities were highly detected in the LF3 isolate. In addition, LP6 showed notable production of *β*-glucosidase. Most strains had high valine arylamidase and leucine arylamidase activities, with relatively low cystine arylamidase activity.

### Evaluation of Antibiotic Susceptibilities of LAB Strains

Table S8 shows antibiotic susceptibility assay results of LAB isolates. The tested LAB strains showed variable patterns of antibiotic susceptibility. All tested LAB isolates were resistant to metronidazole, cefoxitin, streptomycin, ciprofloxacin (except LPa4), amikacin, and vancomycin. On the other hand, they were all susceptible to carbenicillin, clindamycin, erythromycin, and chloramphenicol (except LP1). Most isolates were intermediately resistant to ampicillin except LF3, LPa4, and LP5 (susceptible). In the case of tetracycline, four isolates (i.e., LP1, PA2, LP6, and LS8) were resistant, two (LF3 and PA7) were intermediately resistant, and two (LPa4 and LP5) were susceptible.

The minimal inhibitory concentration (MIC) was also investigated against various antibiotics for multiple LAB strains and corresponding EFSA cut-offs for resistance (Table 6). For ampicillins, the MIC values ranged from 0.047 to 1.5 µg/ml, and all the LAB isolates were below the EFSA cut-off of 2–4 µg/ml. In the case of gentamycin, MICs ranged from 3 to 32 µg/ml, with most strains below the EFSA cut-off, except only LF3 (32 µg/ml) exceeding the EFSA cut-off of 16 µg/ml. Kanamycin and streptomycin displayed consistently high MIC (192–256 µg/ml) and (128–192 µg/ml), respectively, well above the EFSA cut-off of 32–64 µg/ml. Erythromycin MICs ranged from 0.047 to 0.38 µg/ml, all well below the EFSA cut-off of 1 µg/ml. Clindamycin exhibited MIC values for LP1, LP5, and LP6 between 1.5 and 2 µg/ml, broadly aligning with the EFSA cut-off of 2 µg/ml. At the same time, the remaining isolates had MIC values ranging from 1.5 to 2, exceeding the EFSA cut-off of 1 µg/ml. For tetracycline and chloramphenicol, MIC values ranged from 0.025 to 4 µg/ml and 0.016 to 3 µg/ml, below the EFSA cut-off of 4–32 µg/ml and 4–8 µg/ml, respectively.

**Table 6:** Minimal Inhibitory Concentration (MIC) of LAB isolates

Antibiotics	Antibiotic susceptibility test, MIC (µg/ml)												
	LP1	LP5	LP6	EFSA Cut-off value	LF3	EFSA Cut-off value	LPa4	EFSA Cut-off value	PA2	PA7	EFSA Cut-off value	LS8	EFSA Cut-off value
Ampicillin	0.25	0.25	0.125	2	0.047	2	0.75	4	1.5	1.5	4	1	4
Gentamycin	3	6	8	16	32	16	32	32	16	16	16	12	16
Kanamycin	256	256	256	64	192	32	256	64	256	256	64	256	64
Streptomycin	n.r	n.r	n.r	n.r	128	64	128	64	128	96	64	192	32
Erythromycin	0.125	0.25	0.19	1	0.094	1	0.047	1	0.25	0.38	1	0.5	1
Clindamycin	1.5	2	2	2	2	1	2	1	2	2	1	1.5	1
Tetracycline	2	4	2	32	1	8	0.025	4	2	4	8	3	8
Chloramphenicol	0.125	1	1	8	0	4	0.032	4	0.016	0.032	4	3	4

*n.r* Not required, MIC value for the antibiotics recommended by the European Food Safety Authority (EFSA), 2012

## Discussion

The probiotics paradigm has evolved from mere functional food additives to an emerging field of therapeutic interventions [36]. As a result, the discovery of novel probiotic strains has become important under these circumstances. A significant shift in probiotic acquisition was recently observed, transitioning from food-based origins to human sources [2]. This shift led us to study probiotics, particularly in the context of specific diseases, thereby allowing the discovery of health-specific strains with clinical potential. In the present study, we focused on postmenopausal women as a unique reservoir of probiotics to identify and characterize strains that might offer therapeutic potential for this particular group of people.

In our current study, we identified and characterized probiotic strains from the vaginal fluid of healthy postmenopausal women. This approach holds considerable significance in developing probiotics explicitly tailored to the unique health needs of this particular group. By studying this specific population, we aimed to unravel probiotic strains that could address health challenges commonly encountered during the postmenopausal phase. The development of customized probiotics for targeted therapeutic applications has been gaining traction in different disease contexts [37]. Notably, some studies demonstrated promising results of custom probiotics, such as *Lactobacillus*-containing feminine products and *Lactobacilli (L. rhamnosus GR-1* and *L. fermentum* RC-14), which alleviated the urogenital pathogenicity and improved the vaginal ecosystems of the affected individuals [38]. Therefore, our study’s findings provide potential probiotic candidates for postmenopausal women and contribute to the broader understanding of personalized probiotic development for disease management. Considering the background mentioned above, we isolated noble probiotic strains from postmenopausal vaginal fluid as a new source of probiotics. Building on Kim et al*.* (2021), who found decreased species richness but increased diversity in postmenopausal vaginal samples, our current study aims to isolate diverse probiotic strains from these women using a culturomics method optimized for *Lactobacilli* [10]. As a result, various strains were isolated from the vaginal microbiota using the culturomics method, and eight types of lactic acid bacteria were finally isolated through identification. They were first identified through 16S rRNA sequencing technology using a widely used reliable primer set [30], and they come from different lineages, each being unique based on molecular fingerprinting analysis. These strains were further identified by employing biochemical tests. However, the isolated strains were first confirmed for their basic probiotic properties and further tested for functionality and safety.

Initially, we sought to determine the probiotic characteristics of the isolated strains. They were found to produce lactic acid, which is considered a major metabolite produced by *Lactobacillus* species that plays a crucial role in maintaining a healthy vaginal environment [31]. Our study found that all tested isolates could acidify the modified MRS medium, consistent with previously reported findings [31]. The positive health effects of probiotic strains can also be assessed by their ability to withstand the passage through the stomach and gastrointestinal tract and effectively establish colonization in the intestinal environment [39]. The isolated strains exhibited hydrophobicity and auto-aggregation, enabling them to adhere to the intestinal environment and form a protective barrier against the colonization of pathogenic bacteria, complying with previous reports [18]. The isolated LAB strains displayed an excellent adhesion capacity, indicating their easy survivability in the digestive tract. *Lactobacillus rhamnosus* GG is a well-documented, widely used probiotic with known functional characteristics, making it an ideal control strain for evaluating new isolates’ characteristics [40]. The presence of gastric acid in the human gut (pH 2.0) is responsible for the breakdown and elimination of most ingested microbes [19]. Therefore, testing the survival of probiotic strains in gastric acid and bile salt is crucial to ensure their ability to colonize and maintain metabolic activity in the host’s intestine [19, 39]. In this study, all tested LAB isolates could tolerate both gastric acid (except LP1) and bile salt at a concentration of 0.3% and 1% (except LP5), respectively, which confers with previously reported data [41]. Moreover, LAB isolates resisted digestive enzymes, including proteinase K, α-amylase, trypsin, and lysozyme, consistent with previous reports [19].

Beyond the fundamental analysis, the functional characteristics of the isolated LAB strains were investigated following the established guidelines. Candidate LAB strains demonstrated excellent antimicrobial activity against various pathogenic microorganisms, which aligns with the previous findings [41]. Notably, most of the tested pathogens were inhibited, with the highest 98% growth inhibition using the lowest MIC doses, indicating solid antimicrobial activity. LABs also showed lower MIC active killing potentiality against vaginal pathogens, especially *P. bivia*, *G. vaginalis*, and *S. aureus*, consistent with previous studies [42]. Results also showed the ability of the isolated strain to lower the pH due to the production of metabolites like short-chain fatty acids, making the strains valuable for further study. Probiotic strains have been shown to produce bacteriocins, identified using primer-specific PCR, which are naturally occurring proteins and peptides with antimicrobial properties; these function by inducing pore formation or inhibiting cell wall synthesis in target cells. This presents a potential future alternative to antibiotics [43]. In our current study, we noted a similar type of pediocin encoding *pedA*1 gene and other types of class II bacteriocin genes (such as *planA* and *planS*), suggesting their potential use as an antimicrobial agent to inhibit pathogen growth and serve as food preservatives. In addition, probiotic strains displayed their ability to produce different short-chain fatty acids, indicating their potential antibacterial activity by lowering the pH, disrupting cell membranes, inhibiting bacterial enzymes, and modulating the immune response [44]. Short-chain fatty acids (SCFAs) are effective antimicrobial agents that reduce pH and create a hostile environment for various pathogens.

The isolated LAB strains displayed excellent immunomodulation properties, including phagocytosis, effectively inducing the immune cells to engulf and digest the foreign particles, including pathogens, thus protecting the body against infection, which agrees with previous studies [45]. In addition to their excellent antioxidant activities, consistent with the previous findings, the discovered strain may help supplement the human body’s defense mechanisms against oxidative stress, which, when excessive, can damage cells or tissues [46]. Owing to these attributes, probiotics can neutralize oxidative stress, improve the host’s health, and contribute to managing diseases such as cancer and digestive tract disorders [47]. Besides, most isolated LAB strains exhibited excellent nitrite depletion capacity, indicating their potential as starter cultures in fermented foods to prevent nitrite conversion to nitrosamines, which can cause cancer and methemoglobinemia [48]. LAB strains showed their proteolytic activity to hydrolyze milk protein (halos zone), which reduced the allergenicity of dairy products, thereby implying their potential for use in food industries.

Furthermore, LAB isolates were found to produce β-glucosidase. This enzyme is essential in optimizing fermentation processes, improving the nutritional quality of fermented foods [49], and further emphasizing their uses in food-related industries. Moreover, heat-killed LAB strains significantly decreased the expressions of IL-1β, IL-6, and iNOS but not of TNF-α in LPS-treated macrophages. These findings imply that heat-killed LAB strains may stimulate the innate immune system and reduce inflammatory reactions, which agrees with the previously reported findings [50].

The safety of the tested LAB isolates was evaluated, considering their potential use in future drug development. The results revealed that all isolated LAB strains (heat-killed and live, except LPa4) showed no cytotoxicity (viability > 80%) in macrophage cells, which is consistent with the results of a previously published study [47]. The safety of the tested LAB isolates was evaluated using standard physiological characteristics such as biogenic amine synthesis and hemolytic and gelatinase activities. None of the strains produced biogenic amines or H_2_O_2._ In addition, they did not show either hemolytic activity (α or β) or gelatinase activity. None of the tested LAB isolates displayed the presence of biogenic amine genes or virulence genes using the conventional PCR. However, to further confirm the absence of these genes, whole genome sequencing or DNA hybridization studies are required.

The global acceptance of GRAS microorganisms highlights the importance of safety, particularly in *Lactobacillus* spp. used as functional strains, to minimize the risk of genetic transfer, especially conjugated plasmid carrying virulence properties [22]. Most of the LAB strains were susceptible to various antibiotics according to the EFSA food safety guidelines [35], indicating their safety is not transferring antibiotic-resistance genes to pathogenic bacteria, though some were phenotypically resistant. Despite the observed drug resistance, these probiotics exhibit excellent antimicrobial properties against vaginal pathogens and clinical pathogens, along with significant functional properties, making them promising candidates for therapeutic applications. Moreover, the LAB strains displayed the absence of the carcinogenic enzyme β-glucuronidase, which is linked to cancer development [51]; instead, they produced beneficial enzymes consistent with findings from previous studies [22].

The primary goal of this study was to identify probiotic strains from the vaginal fluid of healthy menopausal women and subsequently explore their potential benefits. As the identified strains displayed excellent anti-inflammatory effects, they could be used as a therapeutic intervention, especially considering that postmenopausal women are frequently challenged with inflammatory ailments. In addition, the strain’s considerable antimicrobial activity against vaginal pathogens holds substantial value, particularly given the prevalence of pathogenic bacteria in the vaginal environment of menopausal women. Moreover, these candidates could be employed for both oral and vaginal applications, thereby positioning them as potential strains for health-promoting functional foods and medicines. In pursuing this objective, a comprehensive series of experiments necessary for probiotic development was successfully conducted, which shed light on their potential for broader utilization in disease management.

In summary, we isolated new LAB strains from postmenopausal vaginal microbiota as an emerging source of probiotics and explored their benefits and functional characteristics. However, further in-depth research is necessary to confirm the presence of antimicrobial peptide genes or bacteriocins detected in our study. Moreover, further extensive in-vivo animal experiments are needed to thoroughly investigate their benefits and safety. Despite these limitations, we hope the findings will pave the way for identifying new probiotic strains from untapped sources and their potential application as biotherapeutic agents or advancing the understanding of personalized probiotic development for disease management.

## Conclusion

In this study, lactic acid bacterial strains were isolated and identified from the vaginal microbiota of menopausal women, and their primary and functional characteristics were subsequently explored. The Data demonstrated their potential to be used as biotherapeutic agents for this particular group and in pharmaceutical and nutraceutical sectors for disease management. However, more comprehensive studies are warranted to evaluate their effectiveness and safety.

## Supplementary Information

Below is the link to the electronic supplementary material.

Supplementary file1 (DOCX 3007 kb)
