# Supplementary material for: Isolation of New Strains of Lactic Acid Bacteria from the Vaginal Microbiome of Postmenopausal Women and their Probiotic Characteristics
Source: Curr Microbiol. 2025 Jan 9;82(2):76. doi: 10.1007/s00284-024-04034-8 (PMC11717803; doi:10.1007/s00284-024-04034-8)
Supplement: Supplementary file 1 — Supplementary file1 (DOCX 3007 kb) [file 284_2024_4034_MOESM1_ESM.docx]

**Supplementary data**

**Isolation of new strains of lactic acid bacteria from the vaginal microbiome of postmenopausal women and their probiotic characteristics**

**Indrajeet Barman^1,2†^, Hoonhee Seo^1,2†^, Sukyung Kim^1,2^, Md Abdur Rahim^1,2^, Youjin Yoon^1,2^, Mohammed Solayman Hossain^1,2^, Md Sarower Hossen Shuvo^1,2^, Ho-Yeon Song^1,2*^**

^1^*Department of Microbiology and Immunology, School of Medicine, Soonchunhyang University, Cheonan, 31151, Republic of Korea*.

^2^*Human Microbiome Medical Research Center, Soonchunhyang University, Asan-si, Chungnam 31538, Republic of Korea*

^†^Indrajeet Barman and Hoonhee Seo contributed equally to this study.


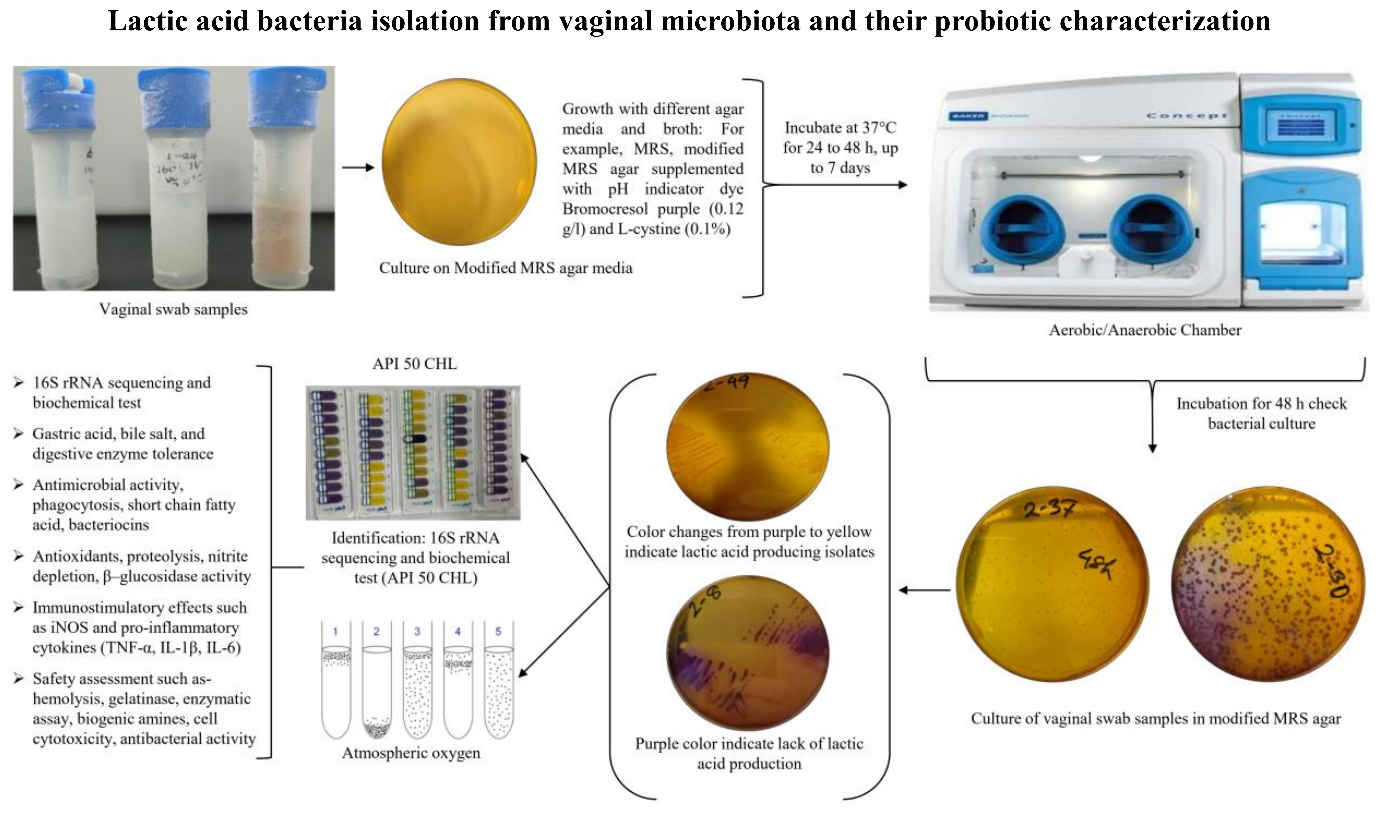


**Fig. S1**. Flow diagram for isolating LAB strains based on culturomics and their characterization. The schematic flow diagram depicts the process of probiotic isolation, identification, and characterization. Postmenopausal vaginal samples were frozen, thawed, cultured on different agar media and broth, and incubated at 37°C in chambers (anaerobic and microaerophilic) for up to 7 days. Subsequently, 8 lactic acid-producing pure bacterial colonies were obtained, subcultured, and sent for 16S rRNA sequencing for identification. They were further identified based on the carbohydrate fermentation pattern. Basic probiotic characterization of the isolated strains included cell surface hydrophobicity, auto-aggregation, adhesion capabilities, survivability in gastric acid, bile salt, and tolerance to digestive enzymes. Subsequently, their functionality was evaluated regarding antimicrobial activity, bacteriocin genes presence, short-chain fatty acids and phagocytosis capabilities, antioxidative activity, rapid depletion of nitrite, and others. Furthermore, their safety profiles were examined, wherein they showed no cytotoxicity in macrophage cell lines. All strains showed negative results for biogenic amine production, displayed no gelatinase or hemolytic activity, and lacked the presence of virulence genes.


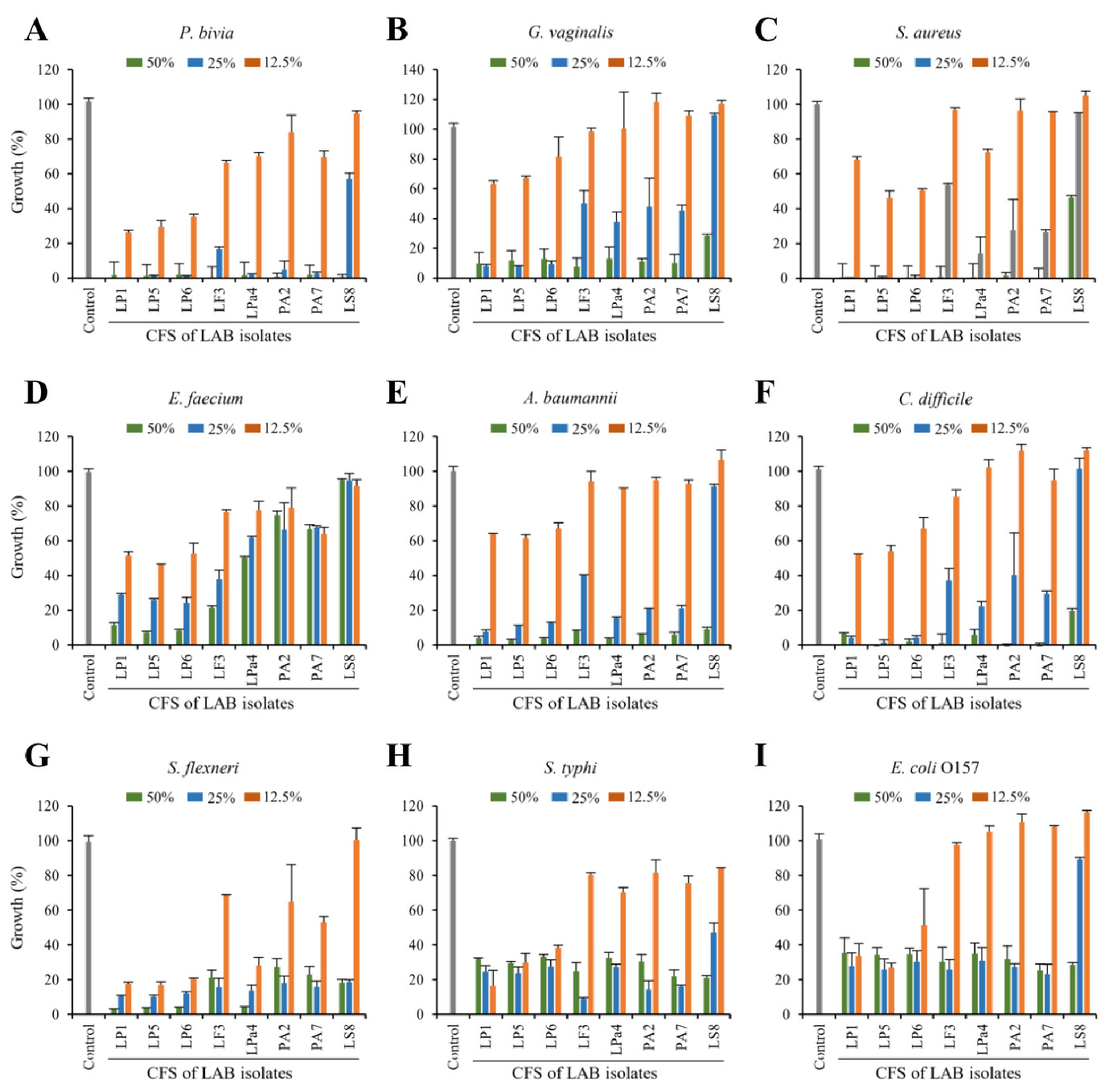


**Fig. S2 Antimicrobial activity of LAB isolates against vaginal and clinical pathogens using microtiter plate turbidimetric assay.** Growth rate of vaginal and clinical pathogens (A) *Prevotella bivia*, (B) *Gardnerella vaginalis*, (C) *Staphylococcus aureus*, (D) *Enterococcus faecium*, (E) *Acinetobacter baumannii*, (F) *Clostridium difficile*, (G) *Shigella flexneri*, (H) *Salmonella typhi*, and (I) *Escherichia coli O157* in the presence of LAB CFS (50%, 25%, and 12.5%) were determined after 24 hours of incubation at 37°C by measuring their OD at 570 nm and calculating their growth rate as a percentage. The highest reduction rate, 98%, was observed for *P. bivia* and *S. aureus* (except LS8) when treated with 50% CFS of LAB isolates. In contrast, E. faecium (except LP5 and LP6), *S. typhi*, and *E. coli* O157 showed the highest growth rates ranging from 12%-95%, 21%-32%, and 28%-35%, respectively. The results are presented as the mean ± standard deviation of triplicate experiments.


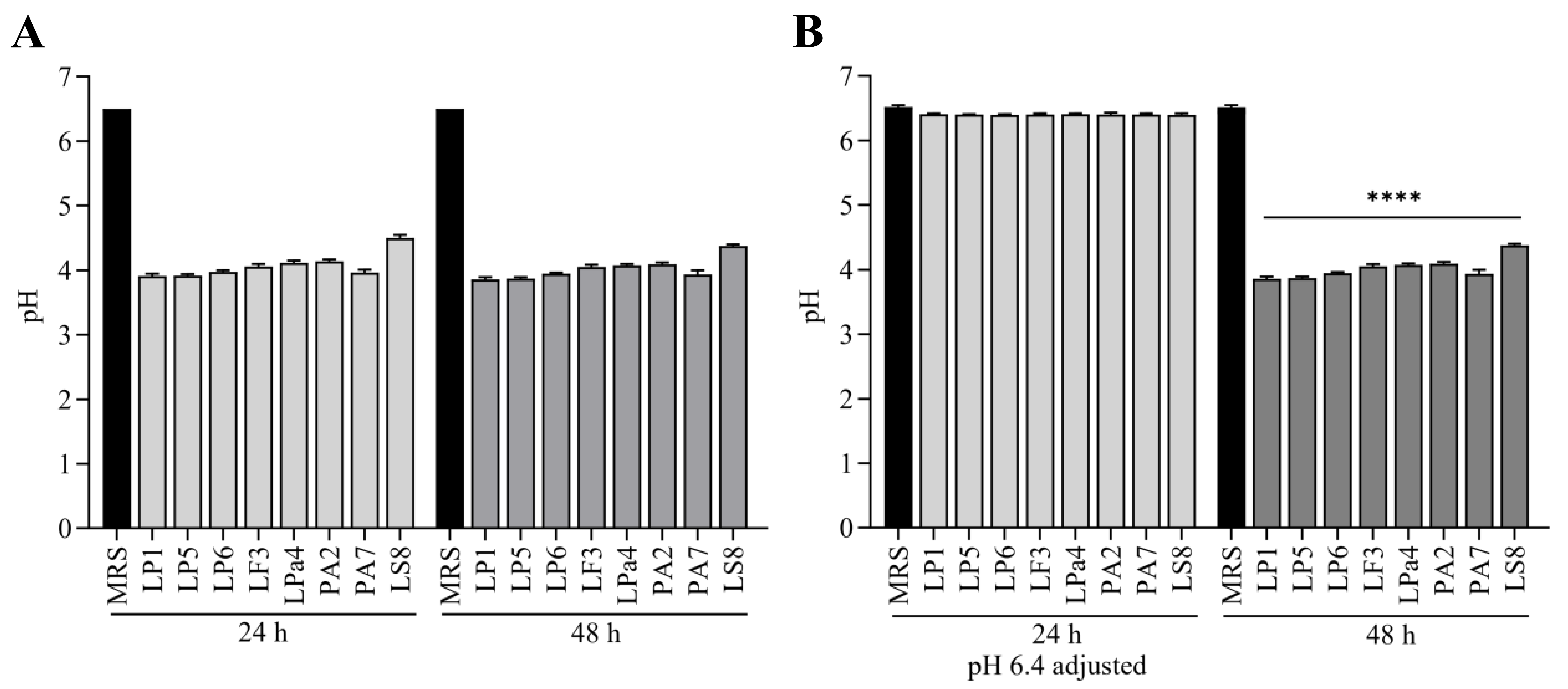


**Fig. S3. Exploring the pH-lowering ability of the isolated lactic acid bacteria (LAB) strains**. The LAB strains were grown on MRS broth for up to 48 h, and their pH was measured at 24 h and 48 h. After 24 h of growth, the pH of the LAB isolates was adjusted to 6.4 using 10M NaOH, and the cultures were further incubated for 48 h to measure decreased pH. The results are presented as the mean ± standard deviation of triplicate experiments. Statistical significance was evaluated by comparing the data to that of the adjusted pH group of 24 h and 48 h using Graph Pad Prism (version 8.0.1) with one-way ANOVA (****, *p* < 0.0001).


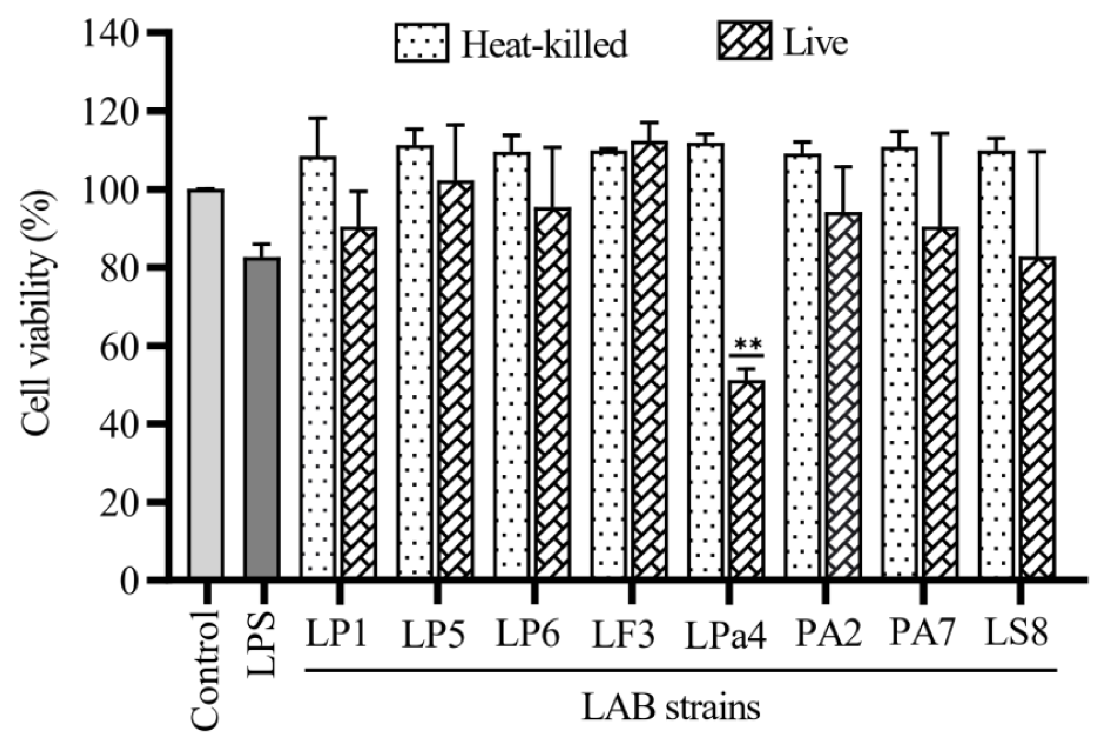


**Fig. S4. Evaluation of cytotoxicity of isolated lactic acid bacteria (LAB) strains**. The impact of isolated LAB strains on macrophage RAW 267.4 cells was explored. The water-soluble tetrazolium salt (WST)-based assay displayed no noteworthy toxicity of cells treated with heat-killed and live LAB strains (1×10^8^ CFU/ml), apart from live LPa4, when compared to untreated RAW cells. The results are presented as the mean ± standard deviation of triplicate experiments. Statistical significance was determined by comparing the obtained data to that of the untreated group using Graph Pad Prism (version 8.0.1) with one-way ANOVA (*, *p* < 0.05; **, *p* < 0.01; ***, *p* < 0.001; and ****, *p* < 0.0001).

**Table S1**. List of bacteria isolated from vaginal samples of postmenopausal women.

| Culture condition | Vaginal samples (VS) | | | | | | | | | | |
| --- | --- | --- | --- | --- | --- | --- | --- | --- | --- | --- | --- |
|  | VS 22 | VS 23 | VS 25 | VS 28 | VS 30 | VS 37 | VS 39 | VS 40 | VS 41 | VS 46 | VS 49 |
|  | Isolates | | | | | | | | | | |
| MRS agar, Bromocresol purple, and L-cystine, 37°C, Microaerophilic | 22MB1, 22MB2 | 23MB1, 23MB2 | 25MB1, 25MB2, 25MB3 | 28MB1, 28MB2, 28MB3 | 30MB1, 30MB2 | 37MB1, 37MB2, 37MB3 | 39MB1, 39MB2 | 40MB1, 40MB2, 40MB3, 40MB4 | 41MB1, 41MB2 | 46MB1, 46MB2 | 49MB1, 49MB2 |
| MRS agar, Bromocresol purple, and L-cystine, 37°C, Anaerobic | - | - | - | - | - | - | - | - | - | - | - |
| MRS agar, 37°C, Microaerophilic | 22M1, 22M2 | 23M1, 23M2 | 25M1, 25M2 | 28M1 | 30M1 | 37M1, 37M2 | 39M1, 39M2 | 40M1 | 41M1, 41M2, 41M3 | 46M1, 46M2 | 49M1 |
| MRS agar, 37°C, Anaerobic | - | - | - | - | - | - | - | - | - | - | - |
| de Manose Sharpe agar, 37°C, Microaerophilic | 22DM1, 22DM2 | 23DM1 | 25DM1, 25DM2 | 28DM1, 28DM2 | 30DM1 | 37DM1, 37DM2, 37DM3 | 39DM1, 39DM2 | 40DM1, 40DM2, 40DM3 | 41DM1, 41DM2 | 46DM1, 46DM2 | 49DM1, 49DM2, 49DM3 |
| de Manose Sharpe agar, 37°C, Anaerobic | - | - | - | - | - | - | - | - | - | - | - |

**Table S2**. Identification of lactic acid bacterial strains based on 16S rRNA gene sequencing.

| Strains ID | Accession number | Closest relative bacterium | Scores (bits) | Gaps | Identity (%) | Ref. NCBI ID |
| --- | --- | --- | --- | --- | --- | --- |
| 22MB2 (LP1) | PQ345789 | *Lactiplantibacillus plantarum* | 2675 (1448) | 0/1450 (0%) | 99% | NR_115605.1 |
|  |  | *Lactiplantibacillus plantarum* | 2675 (1448) | 0/1450 (0%) | 99% | NR_113338.1 |
| 41MB2 (LP5) | PQ345793 | *Lactiplantibacillus plantarum* | 2723 (1474) | 1/1478 (0%) | 99% | NR_115605.1 |
|  |  | *Lactiplantibacillus plantarum* | 2717 (1471) | 1/1478 (0%) | 99% | NR_104573.1 |
| 49MB2 (LP6) | PQ345794 | *Lactiplantibacillus plantarum* | 2736 (1481) | 2/1490 (0%) | 99% | NR_115605.1 |
|  |  | *Lactiplantibacillus plantarum* | 2736 (1481) | 2/1490 (0%) | 99% | NR_113338.1 |
| 39MB1 (LF3) | PQ345791 | *Limosilactobacillus fermentum* | 2686 (1454) | 0/1459 (0%) | 99% | NR_113335.1 |
|  |  | *Limosilactobacillus fermentum* | 2667 (1444) | 0/1450 (0%) | 99% | NR_104927.1 |
| 40MB4 (LPa4) | PQ345792 | *Lacticaseibacillus paracasei* | 2726 (1476) | 2/1486 (0%) | 99% | NR_025880.1 |
|  |  | *Lacticaseibacillus paracasei* | 2723 (1474) | 2/1486 (0%) | 99% | NR_113337.1 |
| 25MB3 (PA2) | PQ345790 | *Pediococcus acidilactici* | 2739 (1483) | 4/1506 (0%) | 99% | NR_042057.1 |
|  |  | *Pediococcus pentosaceus* | 2612 (1414) | 4/1506 (0%) | 99% | NR_042058.1 |
| 28MB1 (PA7) | PQ345795 | *Pediococcus acidilactici* | 2704 (1464) | 3/1481 (0%) | 99% | NR_042057.1 |
|  |  | *Pediococcus pentosaceus* | 2577 (1395) | 3/1481 (0%) | 99% | NR_042058.1 |
| 37M2 (LS8) | PQ345796 | *Latilactobacillus sakei* | 2726 (1476) | 1/1479 (0%) | 99% | NR_042443.1 |
|  |  | *Latilactobacillus sakei* | 2721 (1473) | 0/1473 (0%) | 99% | NR_113821.1 |

| Samples | Isolates | Closest relative non-lactobacillus |
| --- | --- | --- |
| VS 22 | 22MB1, 22M1, 22DM2 | *Enterococcus faecalis* |
|  | 22M2, 22DM1 | *Enterococcus faecium* |
| VS 23 | 23MB1, 23DM1 | *Enterococcus faecalis* |
|  | 23MB2 | *Enterococcus faecium* |
|  | 23M1, 23M2 | *Candida albicans* |
| VS 25 | 25MB1, 25MB2, 25M1, 25M2, 25DM1, 25DM2 | *Enterococcus faecalis* |
| VS 28 | 28MB2, 28M1, 28DM1, 28DM2 | *Enterococcus faecalis* |
|  | 28MB3 | *Enterococcus faecium* |
| VS 30 | 30MB1, 30MB2, 30M1 | *Enterococcus faecalis* |
|  | 30DM1 | *Candida albicans* |
| VS 37 | 37MB1, 37MB2, 37M1, 37DM1, 37DM2, 37DM3 | *Enterococcus faecalis* |
|  | 37MB3 | *Citrobacter freundii* |
| VS 39 | 39MB2, 39M1, 39M2 | *Enterococcus faecalis* |
|  | 39DM1, 39DM2 | *Staphylococcus aureus* |
| VS 40 | 40MB2, 40MB3, 40M1, 40DM3 | *Enterococcus faecalis* |
|  | 40MB1 | *Enterococcus faecium* |
|  | 40DM1 | *Citrobacter freundii* |
|  | 40DM2 | *Staphylococcus aureus* |
| VS 41 | 41M1, 41M2, 41M3, 41DM1, 41DM2 | *Enterococcus faecalis* |
|  | 41MB1 | *Staphylococcus aureus* |
| VS 46 | 46MB2, 46M1, 46DM1, 46DM2 | *Enterococcus faecalis* |
|  | 46MB1 | *Enterococcus faecium* |
|  | 46M2 | *Citrobacter baraakii* |
| VS 49 | 49MB1, 49DM1, 49DM2 | *Enterococcus faecalis* |
|  | 49DM3, 49M1 | *Citrobacter baraakii* |

**Table S3**. List of non-lactobacillus bacterial strains identified in this study.

T**able S4**. Biochemical characterization of lactic acid bacterial strains based on carbohydrate utilization.

| Test Number | | 0 | 1 | 2 | 3 | 4 | 5 | 6 | 7 | 8 | 9 | 10 | 11 | 12 | 13 | 14 | 15 | 16 | 17 | 18 | 19 | 20 | 21 | 22 | 23 | 24 |
| --- | --- | --- | --- | --- | --- | --- | --- | --- | --- | --- | --- | --- | --- | --- | --- | --- | --- | --- | --- | --- | --- | --- | --- | --- | --- | --- |
| Strains | Incubation times | Control | Glycerol | Erythritol | D-arabinose | L-arabinose | D-ribose | D-xylose | L-xylose | D-xylose | Methyl-β-D-xylopyranoside | D-galactose | D-glucose | D-fructose | D-mannose | L-sorbose | L-rhamnose | Dulcitol | Inositol | D-mannitol | D-sorbitol | Methyl-alpha-D-mannopyranoside | Methyl-alpha-D-glucopyranoside | N-acetylglucosamine | Amygdalin | Arbutin |
| LP1 | 24h | - | - | - | - | + | + | - | - | - | - | - | + | + | + | - | - | - | - | + | + | - | - | + | + | + |
|  | 48h | - | - | - | - | + | + | - | - | - | - | + | + | + | + | - | - | - | - | + | + | + | ± | + | + | + |
| LP5 | 24h | - | - | - | - | ± | ± | - | - | - | - | + | + | + | + | - | - | - | - | + | + | ± | - | + | ± | + |
|  | 48h | - | - | - | - | ± | + | - | - | - | - | + | + | + | + | - | - | - | - | + | + | + | ± | + | + | + |
| LP6 | 24h | - | - | - | - | ± | ± | - | - | - | - | + | + | + | + | - | - | - | - | + | + | ± | - | + | ± | + |
|  | 48h | - | - | - | - | ± | + | - | - | - | - | + | + | + | + | - | - | - | - | + | + | + | ± | + | + | + |
| LF3 | 24h | - | - | - | - | - | + | - | - | - | - | - | + | - | - | - | - | - | - | - | - | - | - | - | - | - |
|  | 48h | - | - | - | - | - | + | - | - | - | - | + | + | + | - | - | - | - | - | - | - | - | - | - | - | - |
| LPa4 | 24h | - | - | - | - | - | + | - | - | - | - | + | + | + | + | - | - | - | - | + | - | - | - | + | - | - |
|  | 48h | - | - | - | - | - | + | - | - | - | - | + | + | + | + | + | - | - | - | + | - | - | + | + | - | - |
| PA2 | 24h | - | - | - | - | + | + | + | - | - | - | + | + | + | + | - | + | - | - | - | - | - | - | + | + | + |
|  | 48h | - | - | - | - | + | + | + | - | - | - | + | + | + | + | - | + | - | - | + | - | - | - | + | + | + |
| PA7 | 24h | - | - | - | - | + | + | + | - | - | - | + | + | + | + | - | ± | - | - | - | - | - | - | + | ± | + |
|  | 48h | - | - | - | - | + | + | + | - | - | - | + | + | + | + | - | + | - | - | - | - | - | - | + | + | + |
| LS8 | 24h | - | - | - | - | + | + | + | - | - | - | + | + | + | + | - | + | - | - | - | - | - | - | + | + | + |
|  | 48h | - | - | - | - | + | + | + | - | - | - | + | + | + | + | - | + | - | - | - | - | - | - | + | + | + |
| Test Number | | 25 | 26 | 27 | 28 | 29 | 30 | 31 | 32 | 33 | 34 | 35 | 36 | 37 | 38 | 39 | 40 | 41 | 42 | 43 | 44 | 45 | 46 | 47 | 48 | 49 |
| Strains | Incubation times | Esculin | Salicin | D-cellobiose | D-maltose | D-lactose | D-melibiose | D-saccharose | D-trehalose | Inulin | D-melezitose | D-raffinose | Amidon | Glycogen | Xylitol | Gentiobiose | D-turanose | D-lyxose | D-tagatose | D-fucose | L-fucose | D-arabitol | L-arabitol | Potassium gluconate | Potassium 2-ketogluconate | Potassium 5-ketogluconate |
| LP1 | 24h | + | + | + | + | - | + | + | + | - | + | - | - | - | - | + | + | - | - | - | - | - | - | - | - | - |
|  | 48h | + | + | + | + | ± | + | + | + | ± | + | + | - | - | - | + | + | - | - | - | - | - | - | - | - | - |
| LP5 | 24h | + | + | + | + | + | + | + | + | - | + | - | - | - | - | + | - | - | - | - | - | - | - | - | - | - |
|  | 48h | + | + | + | + | + | + | + | + | ± | + | + | - | - | - | ± | - | - | - | - | - | - | - | ± | - | - |
| LP6 | 24h | + | + | + | + | + | + | + | + | - | + | - | - | - | - | + | - | - | - | - | - | - | - | - | - | - |
|  | 48h | + | + | + | + | + | + | + | + | ± | + | + | - | - | - | ± | - | - | - | - | - | - | - | ± | - | - |
| LF3 | 24h | + | - | - | ± | - | + | + | - | - | - | + | - | - | - | - | - | - | - | - | - | - | - | - | - | - |
|  | 48h | + | - | - | ± | + | + | + | - | - | - | + | - | - | - | - | - | - | - | - | - | - | - | - | - | - |
| LPa4 | 24h | + | + | + | - | + | - | + | + | - | + | - | - | - | - | ± | + | - | + | - | - | - | - | ± | - | - |
|  | 48h | + | + | + | + | + | - | + | + | + | + | - | - | - | - | + | + | - | + | - | - | - | - | + | - | - |
| PA2 | 24h | + | + | + | + | + | + | + | + | - | - | + | - | - | - | - | - | - | + | - | - | - | - | - | - | - |
|  | 48h | + | + | + | + | + | + | + | + | - | - | + | - | - | - | - | - | - | + | - | - | - | - | - | - | - |
| PA7 | 24h | + | + | + | - | - | - | + | + | - | - | - | - | - | - | ± | - | - | + | - | - | - | - | - | - | - |
|  | 48h | + | + | + | - | + | - | + | + | - | - | - | - | + | - | - | - | - | + | - | - | - | - | - | - | - |
| LS8 | 24h | + | + | + | - | - | - | + | + | - | - | - | - | - | - | ± | - | - | + | - | - | - | - | ± | - | - |
|  | 48h | + | + | + | - | + | - | + | + | - | + | - | - | - | - | ± | + | - | + | - | - | - | - | ± | - | - |

**Table S5**. Biogenic amines, H_2_O_2_, gelatinase, and hemolytic activities of lactic acid bacterial strains.

| Test names | Candidate LAB strains | | | | | | | | |
| --- | --- | --- | --- | --- | --- | --- | --- | --- | --- |
|  | LP1 | LP5 | LP6 | LF3 | LPa4 | PA2 | PA7 | LS8 |  |
| Biogenic amines (histidine, ornithine, lysine, and tyrosine) | - | - | - | - | - | - | - | - |  |
| H_2_O_2_ production (TMB+HRP) | - | - | - | - | - | - | - | - |  |
| Gelatinase activity | - | - | - | - | - | - | - | - |  |
| Hemolytic activity (*α*, *β*, *γ*) | *γ* | *γ* | *γ* | *γ* | *γ* | *γ* | *γ* | *γ* |  |

‘-’ indicates negative results. TMB, Tetramethylbenzidine; HRP, Horseradish peroxidase.

**Table S6**. Determining the virulence, vancomycin resistance, and biogenic amine genes of the tested lactic acid bacterial isolates.

| Target genes | | LAB Strains | | | | | | | |
| --- | --- | --- | --- | --- | --- | --- | --- | --- | --- |
|  |  | LP1 | LP5 | LP6 | LF3 | LPa4 | PA2 | PA7 | LS8 |
| Virulence genes | *gel*E (gelatinase) | - | - | - | - | - | - | - | - |
|  | *hyl (*Hyaluronidase) | - | - | - | - | - | - | - | - |
|  | *asa*1 (Aggregation substance) | - | - | - | - | - | - | - | - |
|  | *esp* (Enterococcal surface protein) | - | - | - | - | - | - | - | - |
|  | *cyl*A (Cytolysin (*cylA*) | - | - | - | - | - | - | - | - |
|  | *efa*A (Endocarditis antigen) | - | - | - | - | - | - | - | - |
|  | *ace* (Adhesin of collagen protein) | - | - | - | - | - | - | - | - |
| Vancomycin resistance genes | *van*A (Vancomycin resistance) | - | - | - | - | - | - | - | - |
|  | *van*B Vancomycin resistance) | - | - | - | - | - | - | - | - |
| Biogenic amine genes | *hdcA* (Histidine decarboxylase) | - | - | - | - | - | - | - | - |
|  | *tdcA (*Tyrosine decarboxylase) | - | - | - | - | - | - | - | - |
|  | *odcA* (Ornithine decarboxylase) | - | - | - | - | - | - | - | - |

‘-’ indicates negative results of the interested gene.

**Table S7.** Enzymatic profiles of the isolated lactic acid bacterial strains.

| Enzymes | Enzymes activity | | | | | | | |
| --- | --- | --- | --- | --- | --- | --- | --- | --- |
|  | LP1 | LP5 | LP6 | LF3 | LPa4 | PA2 | PA7 | LS8 |
| Control | 0 | 0 | 0 | 0 | 0 | 0 | 0 | 0 |
| Alkaline phosphatase | 0 | 0 | 0 | 0 | 0 | 0 | 0 | 0 |
| Esterase (C4) | 0 | 0 | 0 | 4 | 0 | 0 | 0 | 0 |
| Esterase Lipase (C8) | 0 | 0 | 0 | 2 | 0 | 0 | 0 | 0 |
| Lipase (C14) | 0 | 0 | 0 | 0 | 0 | 0 | 0 | 1 |
| Leucine arylamidse | 2 | 2 | 4 | 1 | 5 | 5 | 5 | 5 |
| Valine arylamidase | 2 | 1 | 3 | 1 | 5 | 5 | 5 | 5 |
| Cystine arylamidase | 0 | 1 | 2 | 1 | 0 | 0 | 0 | 2 |
| Trypsin | 0 | 0 | 0 | 0 | 0 | 0 | 0 | 0 |
| *α*-chymotrypsin | 0 | 0 | 0 | 0 | 0 | 0 | 0 | 0 |
| Acid phosphatase | 0 | 0 | 0 | 0 | 1 | 0 | 0 | 1 |
| Naphthol-A-S-BI-phosphohydrolase | 2 | 2 | 2 | 3 | 2 | 3 | 3 | 3 |
| *α*-galactosidase | 0 | 0 | 0 | 5 | 0 | 0 | 0 | 0 |
| *β*-galactosidase | 0 | 0 | 0 | 5 | 0 | 0 | 0 | 1 |
| *β*-glucuronidase | 0 | 0 | 0 | 0 | 0 | 0 | 0 | 0 |
| *α*-glucosidase | 0 | 0 | 0 | 4 | 3 | 0 | 0 | 0 |
| *β*-glucosidase | 0 | 1 | 4 | 0 | 0 | 1 | 1 | 1 |
| N-acetyl-*β*-glucosidaminidase | 0 | 0 | 2 | 0 | 0 | 0 | 0 | 0 |
| *α*-mannosidase | 0 | 0 | 0 | 0 | 0 | 0 | 0 | 0 |
| *α*-fucosidase | 0 | 0 | 0 | 0 | 0 | 0 | 0 | 0 |

Numerical values represent the color change intensity: 0, no activity; 5, maximum activity.

**Table S8.** Antibacterial susceptibility testing of the isolated lactic acid bacterial strains

| Antibiotics | LP1 | LP5 | LP6 | LF3 | LPa4 | PA2 | PA7 | LS8 |
| --- | --- | --- | --- | --- | --- | --- | --- | --- |
| Carbenicillin | S | S | S | S | S | S | S | S |
| Clindamycin | S | S | S | S | S | S | S | S |
| Erythromycin | S | S | S | S | S | S | S | S |
| Chloramphenicol | I | S | S | S | S | S | S | S |
| Ampicillin | I | S | I | S | S | I | I | I |
| Tetracycline | R | S | R | I | S | R | I | R |
| Ciprofloxacin | R | R | R | R | I | R | R | R |
| Metronidazole | R | R | R | R | R | R | R | R |
| Cefoxitin | R | R | R | R | R | R | R | R |
| Streptomycin | R | R | R | R | R | R | R | R |
| Amikacin | R | R | R | R | R | R | R | R |
| Vancomycin | R | R | R | R | R | R | R | R |

S, susceptible; I, intermediately resistant; R, resistant.

**Table S9**. List of primers used in this study.

| Gene symbol | Gene name | Primers | Oligonucleotide sequence (5'-3') | PCR conditions | Reference |
| --- | --- | --- | --- | --- | --- |
| *ped* | Pediocin PA-1 | PedproF | CAAGATCGTTAACCAGTTT | (94°C 1 min, 50°C 30 second, 72°C 1 min) × 35 | [1] |
|  |  | Ped 1041R | CCGTTGTTCCCATAGTCTAA |  |  |
| *nisQ* | Nisin | nisF | ATGAGTACAAAGATTTCAACTT | (94°C 45 second, 48°C 30 second, and 72°C 45 second) × 30 | [2] |
|  |  | nisR | TTATTTGCTTACGTGAACGC |  |  |
| *plan W* | Plantaracin W | planW-F | TCACACGAAATATTCCA | (95°C 1 min, 53°C 1 min, 72°C 1 min) × 30 | [3] |
|  |  | planW-R | GGCAAGCGTAAGAAATAAATGAG |  |  |
| *plan S* | Plantaracin S | planS-F | GCCTTACCAGCGTAATGCCC | (93°C 30 second, 60°C 40 second, 72°C 40 second) × 30 | [4] |
|  |  | planS-R | CTGGTGATGCAATCGTTAGTTT |  |  |
| *plan NC8* | Plantaracin NC8 | planNC8-F | GGTCTGCGTATAAGCATCGC | (94°C 30 second, 42°C 1 min, 72°C 1 min) × 30 | [5] |
|  |  | planNC8-R | AAATTGAACATATGGGTGCTTTAAATTCC |  |  |
| *planA* | Plantracin A | planA-F | ATGAAAATTCAAATTAAAGG | (94°C 1 min, 55°C 1 min, 72°C 30 second) × 30 | [6] |
|  |  | planA-R | TTACCATCCCCATTTTTTA |  |  |
| *skgA*1 | Sakacin GA-1 | skgA1F | TTAGAACTACACTGATCGTG | (94°C 30 second, 52 °C 30 second and 72 °C 30 second) × 30 | [7] |
|  |  | skgA1R | TGGAAGAATGAGTACTTGTT |  |  |
| *skgA*2 | Sakacin GA-2 | skgA2F | CGTTACAACAGAACTTCAAG |  |  |
|  |  | skgA2R | TGGAAGAATGAGTACTTGTT |  |  |
| *gelE* | Gelatinase | gel11-F | TATGACAATGCTTTTTGGGAT | (94°C 1 min, 56°C 1 min, 72°C 1 min) × 30 | [8] |
|  |  | gel12-R | AGATGCACCCGAAATAATATA |  |  |
| *hyl* | Hyaluronidase | hyl n1-F | ACAGAAGAGCTGCAGGAAATG |  |  |
|  |  | hyl n2-R | GACTGACGTCCAAGTTTCCAA |  |  |
| *asa1* | Aggregation substance | asa11-F | GCACGCTATTACGAACTATGA |  |  |
|  |  | asa12-R | TAAGAAAGAACATCACCACGA |  |  |
| *esp* | Enterococcal surface protein | esp14-F | AGATTTCATCTTTGATTCTTGG |  |  |
|  |  | esp12-R | AATTGATTCTTTAGCATCTGG |  |  |
| *cylA* | Cytolysin | cyt Ib | ACTCGGGGATTGATAGGC |  |  |
|  |  | cyt IIb | GCTGCTAAAGCTGCGCTT |  |  |
| *efaA* | Endocarditis antigen | efa-AF | GCCAATTGGGACAGACCCTC | (94°C 1 min, 55°C 1 min, 72°C 1 min) × 30 | [9] |
|  |  | efa-AR | CGCCTTCTGTTCCTTCTTTGGC |  |  |
| *ace* | Adhesin of collagen protein | ace-F | GAATTGAGCAAAAGTTCAATCG |  |  |
|  |  | ace-R | GTCTGTCTTTTCACTTGTTTC |  |  |
| *vanA* | Vancomycin resistance | van-AF | TCTGCAATAGAGATAGCCGC | (94°C 1 min, 55°C 1 min, 72°C 1 min) × 30 | [9] |
|  |  | van-AR | GGAGTAGCTATCCCAGCATT |  |  |
| *vanB* | Vancomycin resistance | van-BF | GCTCCGCAGCCTGCATGGACA |  |  |
|  |  | van-BR | ACGATGCCGCCATCCTCCTGC |  |  |
| *hdc* | Histidine decarboxylase | JV16HC | AGATGGTATTGTTTCTTATG | (94°C 30 second, 52°C 30 second, 72°C 2 min) × 30 | [10] |
|  |  | JV17HC | AGACCATACACCATAACCTT |  |  |
| *tdc* | Tyrosine decarboxylase | P2-F | GAYATNATNGGNATNGGNYTNGAYCARG |  |  |
|  |  | P1-R | CCRTARTCNGGNATAGCRAARTCNGTRTG |  |  |
| *odc* | Ornithine decarboxylase | Orn3-F | GTNTTYAAYGCNGAYAARACNTAYTTYGT |  |  |
|  |  | Orn16-R | ATNGARTTNAGTTCRCAYTTYTCNGG |  |  |
| iNOS | iNOS | Forward | CCTCACGCTTGGGTCTTGTT | (95°C 10 second, 55~60°C 10 second, 72°C 30 second) × 39 | [11] |
|  |  | Reverse | GCACAAGGGGTTTTCTTCACG |  |  |
| TNF-α | TNF-α | Forward | AGGCACTCCCCCAAAAGATG |  | [12] |
|  |  | Reverse | CACCCCGAAGTTCAGTAGACAGA |  |  |
| IL-1β | IL-1β | Forward | TGACGGACCCCAAAAGAT |  |  |
|  |  | Reverse | GTGATACTGCCTGCCTGAAG |  |  |
| IL-6 | IL-6 | Forward | CCGGAGAGGAGACTTCACAGAG |  |  |
|  |  | Reverse | TCATTTCCACGATTTCCCAGAG |  |  |
| GAPDH | GAPDH | Forward | CATGGCCTTCCGTGTTCCTAC |  |  |
|  |  | Reverse | TCAGTGGGCCCTCAGATGC |  |  |

TNF-α, tumor necrosis factor-α; IL-β, interleukin-1β; IL-6, interlukin-6; *iNOS*, inducible nitric oxide synthase.

**Table S10.** 16s RNA sequencing results of *Enterococcus faecium* and *Staphylococcus aureus* isolated from clinical vaginal samples of Korean women.

| Strains | Closest relative bacterium | Scores (bits) | Gaps | Identity (%) | Ref. NCBI ID |
| --- | --- | --- | --- | --- | --- |
| *Enterococcus faecium* | *Enterococcus faecium* strain DSM 20477 | 2732 (1479) | 2/1492 (0%) | 99% | NR_114742.1 |
|  | *Enterococcus faecium* strain NBRC 100486 | 2717 (1471) | 2/1486 (0%) | 99% | NR_113904.1 |
|  | *Enterococcus faecium* strain ATCC 19434 | 2712 (1468) | 2/1483 (0%) | 99% | NR_115764.1 |
|  | *Enterococcus durans* strain NBRC 100479 | 2710 (1467) | 2/1486 (0%) | 99% | NR_113900.1 |
|  | *Enterococcus durans* strain JCM 8725 | 2706 (1465) | 2/1483 (0%) | 99% | NR_113257.1 |
| *Staphylococcus aureus* | *Staphylococcus aureus* strain S33 | 2719 (1472) | 1/1488 (0%) | 99% | NR_037007.2 |
|  | *Staphylococcus aureus* strain ATCC 12600 | 2713 (1469) | 1/1488 (0%) | 99% | NR_118997.2 |
|  | *Staphylococcus aureus* strain NBRC 100910 | 2710 (1467) | 1/1477 (0%) | 99% | NR_113956.1 |
|  | *Staphylococcus aureus* subsp. anaerobius strain MVF-7 | 2702 (1463) | 1/1476 (0%) | 99% | NR_036828.1 |
|  | *Staphylococcus simiae* CCM 7213 | 2678 (1450) | 1/1478 (0%) | 99% | NR_043146.1 |

**References**

1. Todorov SD, Ho P, Vaz-Velho M, Dicks LMT. Characterization of bacteriocins produced by two strains of Lactobacillus plantarum isolated from Beloura and Chouriço, traditional pork products from Portugal. Meat Science. 2010;84(3):334-43. doi: <https://doi.org/10.1016/j.meatsci.2009.08.053>.

2. Kruger MF, Barbosa MdS, Miranda A, Landgraf M, Destro MT, Todorov SD, et al. Isolation of bacteriocinogenic strain of Lactococcus lactis subsp. lactis from rocket salad (Eruca sativa Mill.) and evidences of production of a variant of nisin with modification in the leader-peptide. Food Control. 2013;33(2):467-76. doi: <https://doi.org/10.1016/j.foodcont.2013.03.043>.

3. Holo H, Jeknic Z, Daeschel M, Stevanovic S, Nes IF. Plantaricin W from Lactobacillus plantarum belongs to a new family of two-peptide lantibiotics. Microbiology (Reading). 2001;147(Pt 3):643-51. doi: 10.1099/00221287-147-3-643.

4. Stephens SK, Floriano B, Cathcart DP, Bayley SA, Witt VF, Jiménez-Díaz R, et al. Molecular Analysis of the Locus Responsible for Production of Plantaricin S, a Two-Peptide Bacteriocin Produced by Lactobacillus plantarum LPCO10. 1998;64(5):1871-7. doi: doi:10.1128/AEM.64.5.1871-1877.1998.

5. Maldonado A, Ruiz-Barba JL, Jiménez-Díaz R. Purification and Genetic Characterization of Plantaricin NC8, a Novel Coculture-Inducible Two-Peptide Bacteriocin from Lactobacillus plantarum NC8. 2003;69(1):383-9. doi: doi:10.1128/AEM.69.1.383-389.2003.

6. Doulgeraki AI, Paraskevopoulos N, Nychas GJ, Panagou EZ. An in vitro study of Lactobacillus plantarum strains for the presence of plantaricin genes and their potential control of the table olive microbiota. Antonie Van Leeuwenhoek. 2013;103(4):821-32. doi: 10.1007/s10482-012-9864-2.

7. Todorov SD, Rachman C, Fourrier A, Dicks LMT, van Reenen CA, Prévost H, et al. Characterization of a bacteriocin produced by Lactobacillus sakei R1333 isolated from smoked salmon. Anaerobe. 2011;17(1):23-31. doi: <https://doi.org/10.1016/j.anaerobe.2010.01.004>.

8. Vankerckhoven V, Autgaerden TV, Vael C, Lammens C, Chapelle S, Rossi R, et al. Development of a Multiplex PCR for the Detection of asa1, gelE, cylA, esp, and hyl Genes in Enterococci and Survey for Virulence Determinants among European Hospital Isolates of Enterococcus faecium. 2004;42(10):4473-9. doi: doi:10.1128/jcm.42.10.4473-4479.2004.

9. Martín-Platero AM, Valdivia E, Maqueda M, Martínez-Bueno M. Characterization and safety evaluation of enterococci isolated from Spanish goats' milk cheeses. International Journal of Food Microbiology. 2009;132(1):24-32. doi: 10.1016/j.ijfoodmicro.2009.03.010.

10. De las Rivas B, Marcobal Á, Muñoz R. Improved multiplex-PCR method for the simultaneous detection of food bacteria producing biogenic amines. FEMS Microbiology Letters. 2005;244(2):367-72. doi: 10.1016/j.femsle.2005.02.012 %J FEMS Microbiology Letters.

11. Gao X, Zeng R, Ho CT, Li B, Chen S, Xiao C, et al. Preparation, chemical structure, and immunostimulatory activity of a water-soluble heteropolysaccharide from Suillus granulatus fruiting bodies. Food Chem X. 2022;13:100211. doi: 10.1016/j.fochx.2022.100211.

12. Song YR, Lee CM, Lee SH, Baik SH. Evaluation of Probiotic Properties of Pediococcus acidilactici M76 Producing Functional Exopolysaccharides and Its Lactic Acid Fermentation of Black Raspberry Extract. Microorganisms. 2021;9(7). doi: 10.3390/microorganisms9071364.
